# Modeling the Selectivity of Hydrotalcite-Based Catalyst in the Propane Dehydrogenation Reaction

**DOI:** 10.1021/acs.iecr.3c01076

**Published:** 2023-10-09

**Authors:** Giovanni Festa, Palma Contaldo, Marco Martino, Eugenio Meloni, Vincenzo Palma

**Affiliations:** Department of Industrial Engineering, University of Salerno, Via Giovanni Paolo II 132, 84084 Fisciano, SA, Italy

## Abstract

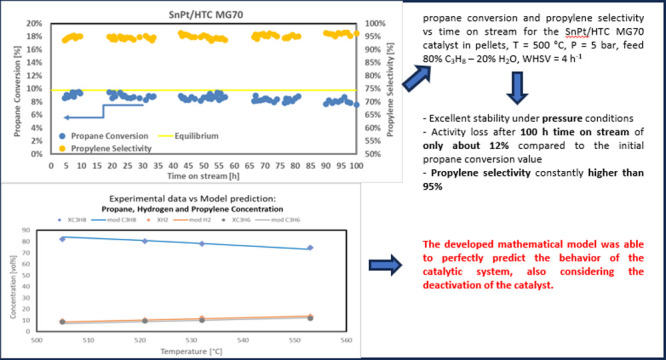

The propylene production processes currently used in the petrochemical industry (fluid catalytic cracking and steam cracking of naphtha and light diesel) are unable to meet the increase of propylene demand for industrial applications. For this reason, alternative processes for propylene production have been investigated, and among the others, the propane dehydrogenation (PDH) process, allowing the production of propylene as a main product, has been industrially implemented (e.g., Catofin and Oleflex processes). The main drawback of such processes is closely linked to the high temperature required to reach a sustainable propane conversion that affects catalyst stability due to coke formation on the catalyst surface. Accordingly, the periodic regeneration of the catalytic bed is required. In this work, the performance in the PDH reaction of different Sn–Pt catalysts, prepared starting by alumina- and hydrotalcite-based supports, is investigated in terms of propane conversion and selectivity to propylene in order to identify a more stable catalyst than the commercial ones. The experimental tests evidenced that the best performance was obtained using the catalyst prepared on commercial pellets of hydrotalcite PURALOX MG70. This catalyst has shown, under pressure conditions of 1 and 5 bar (in order to evaluate the potential future application in integrated membrane reactors), propane conversion values close to the thermodynamic equilibrium ones in all of the investigated temperature ranges (500–600 °C) and the selectivity was always higher than 95%. So, this catalyst was also tested in a stability run, performed at 500 °C and 5 bar: the results highlighted the loss of only 12% in the propane conversion with no changes in the selectivity to propylene. Properly designed experimental tests have also been performed in order to evaluate the kinetic parameters, and the developed mathematical model has been optimized to effectively describe the system behavior and the catalyst deactivation.

## 1 Introduction

In recent times, the increase of polypropylene demand has led propylene to be one of the most widespread feedstocks in the petrochemical and organic industries (about 6% per year).^1^ About 30% of the propylene produced in the world is used for purposes different from polymerization, including the production of propylene oxide (8%), oxo alcohols (8%), acrylonitrile (7%), cumene (4%), and other chemicals.^1^ The propylene production processes currently used in the petrochemical industry involve fluid catalytic cracking and steam cracking of naphtha and light diesel.^2^ However, these production methods are currently unable to meet the increase of propylene demand for industrial applications due to the rapid depletion of fossil energy.^2^ One more disadvantage of these processes is that propylene is only one of the products and not the main one. For this reason, alternative processes for propylene production have been investigated, and among the others, the propane dehydrogenation (PDH) process, allowing us to produce propylene as a main product, has been industrially implemented (e.g., Catofin and Oleflex processes).^3^ In general, PDH is an endothermic reaction, so requiring high operating temperatures for obtaining high propylene yields and the use of a proper catalytic formulation can guarantee propylene selectivity values higher than 90%.^3^ However, under these harsh conditions, the thermal cracking reactions leading to coke and light alkanes may occur, resulting in propylene yield decrease and higher catalyst deactivation rate.^4,5^ Therefore, the coke formed by the various side reactions that occur during PDH, such as cracking and hydrogenolysis, can easily deposit on the Pt active sites^6−8^ and frequent regeneration steps of the catalyst are necessary. As a consequence, to make PDH a competitive process at an industrial level, it is therefore essential to identify a catalytic formulation able to provide high performance in terms of propylene yield and selectivity while suppressing all of those side reactions that lead to the coke formation and, consequently, to the catalyst deactivation. Intensive research was carried out in order to enlarge the activity cycle and thus to increase the time between regenerations by modifying catalytic formulation in terms of both active species and support or operating conditions. Regarding the catalyst, a possible solution is the addition of a metallic promoter, such as tin, to the catalytic formulation. It is widely reported that the addition of tin in Pt-based catalysts can suppress few side reactions that lead to coke formation, thus improving the propylene selectivity and the catalyst stability.^6−12^ Alumina support is widely reported for the PDH reaction. Its large surface area and low cost make it one of the best usable supports in the industrial catalysis.^13^ However, this support has strongly acidic sites that favor the coke formation in this kind of reaction, thus negatively affecting the catalytic performance. Doping alumina with basic oxides is an excellent way to manipulate the active site acidity. In particular, magnesium oxides have proved to be very useful for this purpose, as Mg-doped alumina showed not only a decrease in the support acidity but also an improvement in the Sn interaction with both Pt and support.^14^ These improved interactions led to a greater amount of Sn in its oxidized states, thus decreasing aggregates of metal particles and improving catalytic performance.^14^ Among the supports reported in literature studies, calcined hydrotalcite proved to be very interesting for PDH application. The excellent catalytic performance shown by this support is mainly due to the formation of a mixed Mg(Al)O oxide upon calcination of the hydrotalcite structure. This mixed oxide can be regarded as a defect-rich aluminum-containing magnesium oxide, which are basic sites as well as thermally stable. Furthermore, this support has Al cations on its surface, which can improve the Pt particle dispersion. Moreover, the calcined hydrotalcite has a large specific surface area and much higher resistance to sintering.^15−17^ Consequently, in this work, the performance in the PDH reaction of different Sn–Pt catalysts, prepared starting with alumina- and hydrotalcite-based supports, is investigated in terms of propane conversion and selectivity to propylene in order to identify a more stable catalyst than the commercial ones. To this aim, a comprehensive study of catalytic behavior is essential, and accordingly, a dedicated experimental campaign has been performed. Regarding the operating conditions, the addition of steam can act as a heat carrier toward the catalytic system, as well as could suppress coke deposition;^18^ moreover, it results in a dilution of the system, thermodynamically promoting the propane conversion. On the other hand, the presence of steam could generate a reforming reaction, reducing selectivity toward propylene. So, the experimental tests have been performed by considering the presence of steam in the reacting feed. The starting point has been the optimized steam content in the feed obtained in previous research of our group.^18^

One more possibility for the intensification of the PDH process is the application of integrated membrane reactors, as this reactor configuration has the potential to replace the currently used fixed bed reactors, which necessarily require continuous regeneration cycles.^19^ The use of hydrogen selective membranes allows the continuous removal of hydrogen from the system, thus favoring the thermodynamics of the reaction, according to the Le Chatelier principle, toward an increase in the product formation and, consequently, with an increase in reagent conversion. This phenomenon would allow the possibility of lowering the operating temperatures in an integrated membrane reactor, making the compromise between conversion and selectivity no longer necessary and considerably reducing the coke formation.^20^ On the other hand, the use of the hydrogen selective membrane can lead to extremely low H_2_ partial pressures in the reaction mixture, negatively affecting the selectivity.^19^ Moreover, in some studies, it has been reported that hydrogen selective membranes, at temperatures above 250 °C, suffer greatly from the formation of coke, which, by depositing on the surface of the membrane, inhibits the dissociation of hydrogen, reducing its ability to permeation.^21^ Furthermore, it must be considered that for the membrane correct functioning, quite high operating pressures are necessary with a consequent worsening of the process performance (PDH reaction proceeds with an increase in the moles number and therefore it is favored at low pressures). Therefore, also experimental tests under pressure conditions of 5 bar have been performed in order to assess the possibility of intensifying the process by adding a membrane. The experimental tests evidenced that the best performance was obtained by using the catalyst prepared on commercial pellets of hydrotalcite PURALOX MG70. This catalyst has shown, under pressure conditions of 1 and 5 bar (in order to evaluate the potential future application in integrated membrane reactors), propane conversion values close to the thermodynamic equilibrium ones in all of the investigated temperature ranges (500–600 °C) and the selectivity was always higher than 95%. So, this catalyst was also tested in stability run, performed at 500 °C and 5 bar: the results highlighted the loss of only 12% in the propane conversion with no changes in the selectivity to propylene. Properly designed experimental tests have also been performed in order to evaluate the kinetic parameters, and the developed mathematical model has been optimized to effectively describe the system behavior and the catalyst deactivation. In this way, the experimental findings and the mathematical model derived in this work may provide essential tools for the catalytic dehydrogenation of concentrated propane at a relatively high pressure and low temperature.

## 2 Experimental Section

### 2.1 Catalyst Preparation

Three supports were used for the tests: γ-alumina (γ-Al_2_O_3_), in powder form by SASOL, commercial hydrotalcite (HTC) in powder form by Sigma-Aldrich, and commercial hydrotalcite PURALOX MG70 (HTC MG70) in pellet form (5 × 5 mm^2^) by SASOL. The metal salt precursors platinum(IV) tetrachloride 99% and tin(II) chloride 98% were purchased, respectively, by Carlo Erba and Sigma-Aldrich. Distilled water was purchased by BestChemical, while ethanol was purchased by Carlo Erba. Catalysts were prepared by sequential wet impregnation of the supports, based on the pore volume, with an ethanol solution of tin chloride and aqueous solution of platinum chloride, with a nominal metal loading of 0.7 wt % for Sn and 0.5 wt % for Pt. After each impregnation, the samples were dried for 24 h at 80 °C, dried again for 2 h at 120 °C, and finally calcined for 3 h at 600 °C.

### 2.2 Catalyst Characterization

Both supports and catalysts were characterized by means of a series of analytical techniques. X-ray diffractogram analyses were performed by using an X-ray powder diffractometer (model D8-Advance; Bruker) with a Cu-sealed tube source. Samples were placed in the holder and flattened with a glass slide to ensure a good surface texture. The analyses were performed under the following conditions: Ni-filtered Cu Kα radiation, λ = 1.54 Å, 2θ angle ranging from 20 to 80° with a scan rate of 0.5 s/step, and a step size of 0.0814°. Nitrogen physisorption at 77 K (Quantachrome Instruments, mod. Nova 1200e) was used for the determination of the adsorption–desorption isotherm curves and the evaluation of the specific surface area by using the Brunauer–Emmett–Teller (BET) method and porosimetric characteristics by using the Barrett–Joyner–Halenda (BJH) method. The catalyst prepared in pellet shape has been characterized also by means of Hg penetration technique, with “PASCAL 140” and “PASCAL 240” (Thermo Finnigan Instruments). Raman spectroscopy on the spent catalysts was performed with inVia Raman Microscope Renishaw, equipped with a 514 nm Ar ion laser at 25 mW. The thermogravimetric analysis (TGA) on spent catalysts was performed in an air flow rate of 100 N mL min^–1^ from 25 to 1000 °C with a heating ramp of 10 °C min^–1^ and analyzed by a Q600 connected to a quadrupole-mass spectrometer detector Discovery MS TA Instrument. The active phase reducibility was evaluated by hydrogen temperature-programmed reduction (H_2_-TPR) performed in a tubular reactor with an internal diameter of 14 mm loaded with 5 g of catalysts, feeding a 5 vol % of H_2_ in an Ar gas mixture (flow rate of 100 N mL min^–1^ g_cat_^–1^) and in the temperature range of 25–680 °C (heating ramp of 10 °C min^–1^). The catalyst acidity and basicity were measured by CO_2_ temperature-programmed desorption (CO_2_-TPD). After the H_2_-TPR test, the catalysts underwent an aging process under 10 vol % of H_2_ in an Ar gas mixture (flow rate of 100 N mL min^–1^ g_cat_^–1^) at 580 °C for 5 h to simulate the catalyst condition during the reaction experiments. The samples were then treated in 10 vol % of CO_2_ in an Ar gas mixture at 50 °C for 1 h followed by Ar purge. The TPD was performed by flowing Ar in the temperature range of 25–680 °C (heating ramp of 10 °C min^–1^). After the CO_2_-TPD test, the catalyst was cooled to determine the metal dispersion by CO pulse chemisorption at room temperature. A series of CO pulses were performed with an interval of 2 min until the CO signal of the pulses reached a steady-state value feeding 1.5 vol % of CO in Ar (100 N mL min^–1^ g_cat_^–1^). We have assumed a CO-to-surface-metal-atom ratio of 1:1 and a surface area per surface Pt atom of 0.0841 nm^2^/atom. These tests have been performed on fresh and regenerated (after the stability test) catalysts. For all of these tests, a Pfeiffer OMNISTAR mass spectrometer was used as an analysis system. The chemical composition of the samples was determined by X-ray fluorescence (XRF) spectrometry in a ThermoFisher QUANT’X EDXRF spectrometer equipped with a Rh standard tube as the source of radiation.

### 2.3 Catalytic Activity Tests

Before activity tests, the catalysts were activated in situ by the following procedure:

(1) reduction with a stream composed of 5 vol % of H_2_ in N_2_ from room temperature up to 600 °C with subsequent isotherm of 1 h;
(2) oxidation with a stream composed of 5 vol % O_2_ in N_2_ at 600 °C for 1 h;
(3) reduction in 5 vol % H_2_ in a N_2_ flow at 600 °C for 1 h.

For each procedure, a total volumetric flow rate equal to 1000 N mL min^–1^ g_cat_^–1^ was used. The catalytic activity was evaluated in terms of propane conversion and propylene selectivity and yield with the following equation
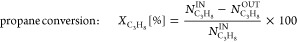
1
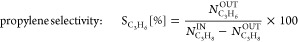
2in which *N*_*i*_^IN^ and *N*_*i*_^OUT^ are the molar flow rates.

The activity tests were performed in a stainless steel tubular reactor with an internal diameter of 1.4 cm and a length of 50 cm, loaded with 13.5 g of catalyst, under atmospheric pressure in the operating temperature range of 500–600 °C at a w8 hly space velocity (WHSV), calculated as follows, of 8 h^–1^ for the powder form catalysts and 4 h^–1^ for the pellet form catalyst with a feed flow composed of propane and steam (C_3_H_8_/H_2_O molar ratio = 4:1)
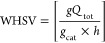
3In which *gQ*_tot_ is the mass flow rate.

For the pellet form catalyst, an activity test under a pressure of 5 bar was also performed in the temperature range of 480–550 °C at a WHSV of 4 h^–1^. The stability test was performed on the pellet form catalyst at a pressure of 5 bar at a fixed temperature of 500 °C with a WHSV of 4 h^–1^. The reactor outlet stream was dried through a refrigerator Julabo F12 and sent to a gas chromatograph Agilent Technologies 7820A, equipped with a flame ionization detector (FID) and a thermoconductivity detector (TCD), evaluating the molar fraction of propane, propylene, hydrogen, CO, CO_2_, methane, ethane, 1-butene, iso-butane, and *n*-butane.

### 2.4 Modeling of the Catalytic Activity

The proposed kinetic scheme for the PDH reaction system is summarized in Table 1.

**Table 1:** Proposed Kinetic Scheme^22^

reaction	kinetic equation
R1. Propane Dehydrogenation	
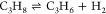	
	*k*_1_ = *k*_01_e^((−*E*_a1_/*R*) × ((1/*T*) – (1/*T*_0_)))^
	*K*_C_3_H_6__ = *K*_0_e^((−Δ*H*/*R*) × ((1/*T*) – (1/*T*_0_)))^
R2. Propane Cracking	
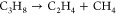	–*r*_2_ = *k*_2_P_C_3_H_8__
	*k*_2_ = *k*_02_e^((−*E*_a2_/R) × ((1/*T*) – (1/*T*_0_)))^
R3. Ethylene Hydrogenation	
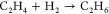	–*r*_3_ = *k*_3_*P*_C_2_H_4_PH_2__
	*k*_3_ = *k*_03_e^((−*E*_a3_/R) × ((1/*T*) – (1/*T*_0_)))^

To limit the loss of activity, steam is supplied as a diluent in the reactant stream that interacts with the catalyst to decrease the formation of coke. The role of steam is to inhibit the cracking reactions and to perform gasification of the coke on the surface of the catalyst; in this way, the catalyst improved in stability and in life. Steam can react, so reforming and water gas shift reactions may occur, and these secondary reactions have also been considered with related kinetic expressions.

The following procedure was used to estimate the kinetic parameters of the main side reactions, as well as the parameters of the deactivation model.

The governing differential equation for the isothermal plug flow reactor employed in the experiments is
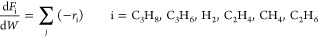
7where *F*_i_ is the flow rate of component i, *W* is the weight of the catalyst, and *r*_i_ is the reaction rate for component i; the sum covers all reactions *j* leading to the formation and/or disappearance of component i.

This equation system, with initial conditions corresponding to the reactor feed, has been solved simultaneously by using the ODEs Numerical Solution Method (Explicit Euler) to obtain the outlet gas composition. The optimum parameter estimation was obtained by minimizing the difference between the experimental and model results using the sum of the root-mean-square error, defined as the objective function OF as follows
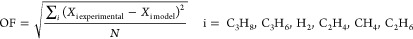
8where i is the number of components of the reaction gas mixture and *N* is the number of experimental points. The screening test results at conditions that are far from the thermodynamic equilibrium were used in order to assume differential reaction conditions with negligible heat and mass transfer effects.

The equilibrium constant *K*_eq_ (Pa) for propane dehydrogenation is given by eq 8(22), which is dependent on the temperature where the reference pressure P0 is the atmospheric pressure expressed in Pa and the temperature is in K
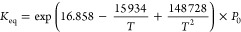
9

Properly designed experimental tests were performed for the determination of the kinetic parameters, as summarized in Table 2.

**Table 2:** Operating Conditions for the PDH Reaction Set

parameter	value	UoM	description
*Q*_tot in_	311	N mL min^–1^	total inlet volumetric flow rate
	624		
	936		
	1872		
	2807		
*F*_tot in_	1.39 × 10^–2^	mol min^–1^	total inlet molar flow rate
	2.78 × 10^–2^		
	4.17 × 10^–2^		
	8.35 × 10^–2^		
	12.5 × 10^–2^		
*P*	5	bar	pressure
*T*	480–550	°C	reaction temperature
WHSV	4–8–12–24–36	h^–1^	weight hourly space velocity
*W*_cat_	8.1	g	catalyst weight

As a result, the optimization of the parameters (in terms of pre-exponential factors *K*_0i_ and activation energies *E*_ai_ for each kinetic constant and adsorption constants) was achieved.

### 2.5 Deactivation of the Catalyst

Since the catalytic system involved in the propane dehydrogenation suffers from an unavoidable deactivation due to several phenomena (coke deposition, active metal sintering), a mathematical model able to predict the activity loss of catalyst was required. In a widely accepted point of view, catalyst deactivation was strictly related to the coke deposition, which of course is affected by operating conditions. The proposed scheme for developing the model with also the deactivation of the catalyst is reported in Table 3.

**Table 3:** Proposed Reaction Scheme with the Catalyst Deactivation

reaction	kinetic equation
R1. Propane Dehydrogenation	
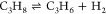	
	γ_1_ = γ_01_e^((−*E*_aγ_/*R*) × ((1/*T*) – (1/*T*_0_)))^
	*k*_1_ = *k*_01_e^((−*E*_a1_/*R*) × ((1/*T*) – (1/*T*_0_)))^
	*K*_C_3_H_6__ = *K*_0_e^((−Δ*H*/*R*) × ((1/*T*) – (1/*T*_0_)))^
R2. Propane Cracking	
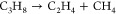	–*r*_2_ = *k*_2_P_C_3_H_8__
	*k*_2_ = *k*_02_e^((−*E*_a2_/R) × ((1/*T*) – (1/*T*_0_)))^
R3. Ethylene Hydrogenation	
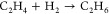	–*r*_3_ = *k*_3_*P*_C_2_H_4_PH_2__
	*k*_3_ = *k*_03_e^((−*E*_a3_/R) × ((1/*T*) – (1/*T*_0_)))^
R4. Coke Formation	
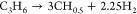	(d*C*_C_/d*t*) = *k*_1C_(*C*_max_ – *C*_m_)^2^ + *k*_2C_

	*C*_M_ = *k*_2C_ × *t*
	*k*_1C_ = *k*_01C_e^((−*E*_a1C_/R) × ((1/*T*) – (1/*T*_0_)))^
	*k*_2C_ = *k*_02C_e^((−*E*_a2C_/R) × ((1/*T*) – (1/*T*_0_)))^

To optimize the mathematical model, three different deactivation models, listed in Table 4, have been evaluated. The OF value has been calculated for each of the next described models, and the model selection criterion is to choose the model that minimizes the OF value.

**Table 4:** Different Deactivation Models for the PDH Rection Set

deactivation model	
D1	*a* = (1 – γ_1_*C*_m_)^2^
D2	*a* = (1 – γ_1_*C*_m_) + γ_2_(*C*_m_/*C*_m_ + *C*_M_)
D3	*a* = (1 – γ_1_*C*_m_) + γ_2_*C*_m_e^(−γ3(*C*_M_/*C*_m_))^

## 3 Results and Discussion

### 3.1 Characterization of the Fresh Catalytic Samples

**Figure 1:**
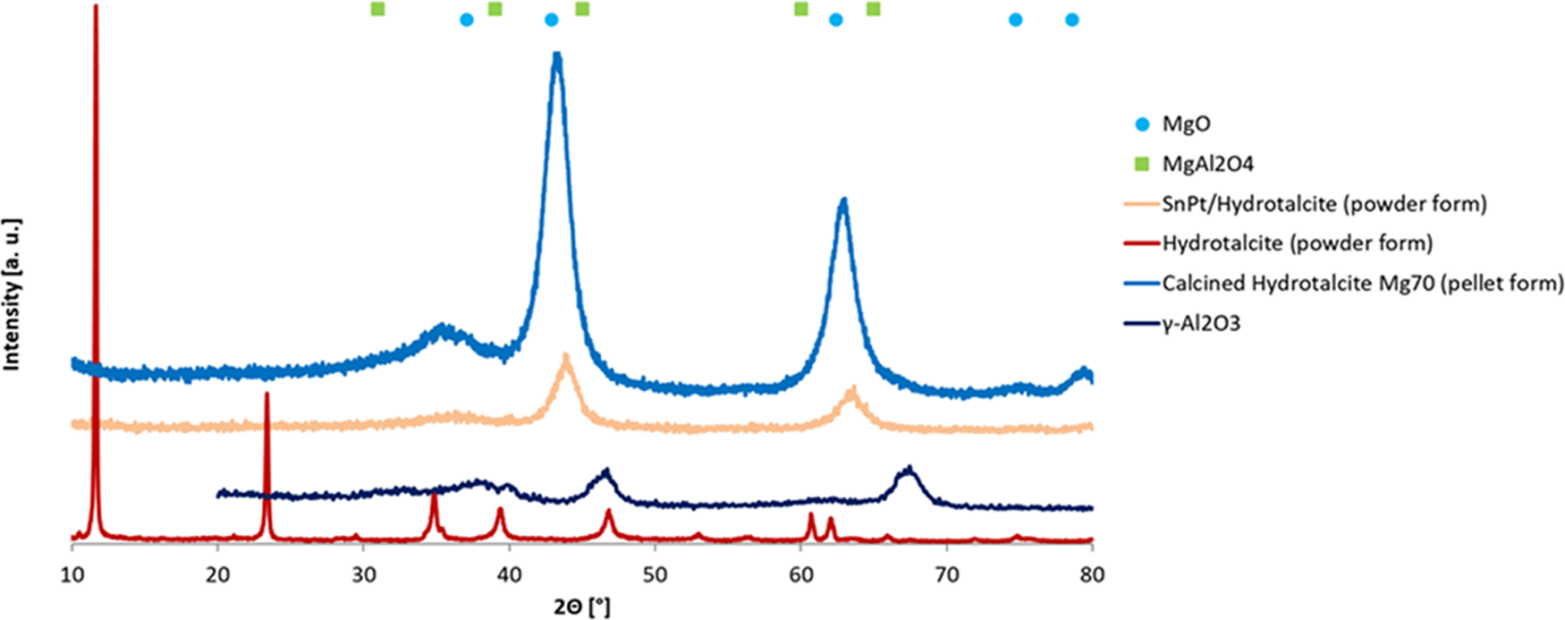
XRD patterns for the hydrotalcite samples.

Figure 1 shows XRD patterns for the hydrotalcite samples, compared with those of MgO, MgAl_2_O_4_, hydrotalcite, and γ-Al_2_O_3_.

**Figure 2:**
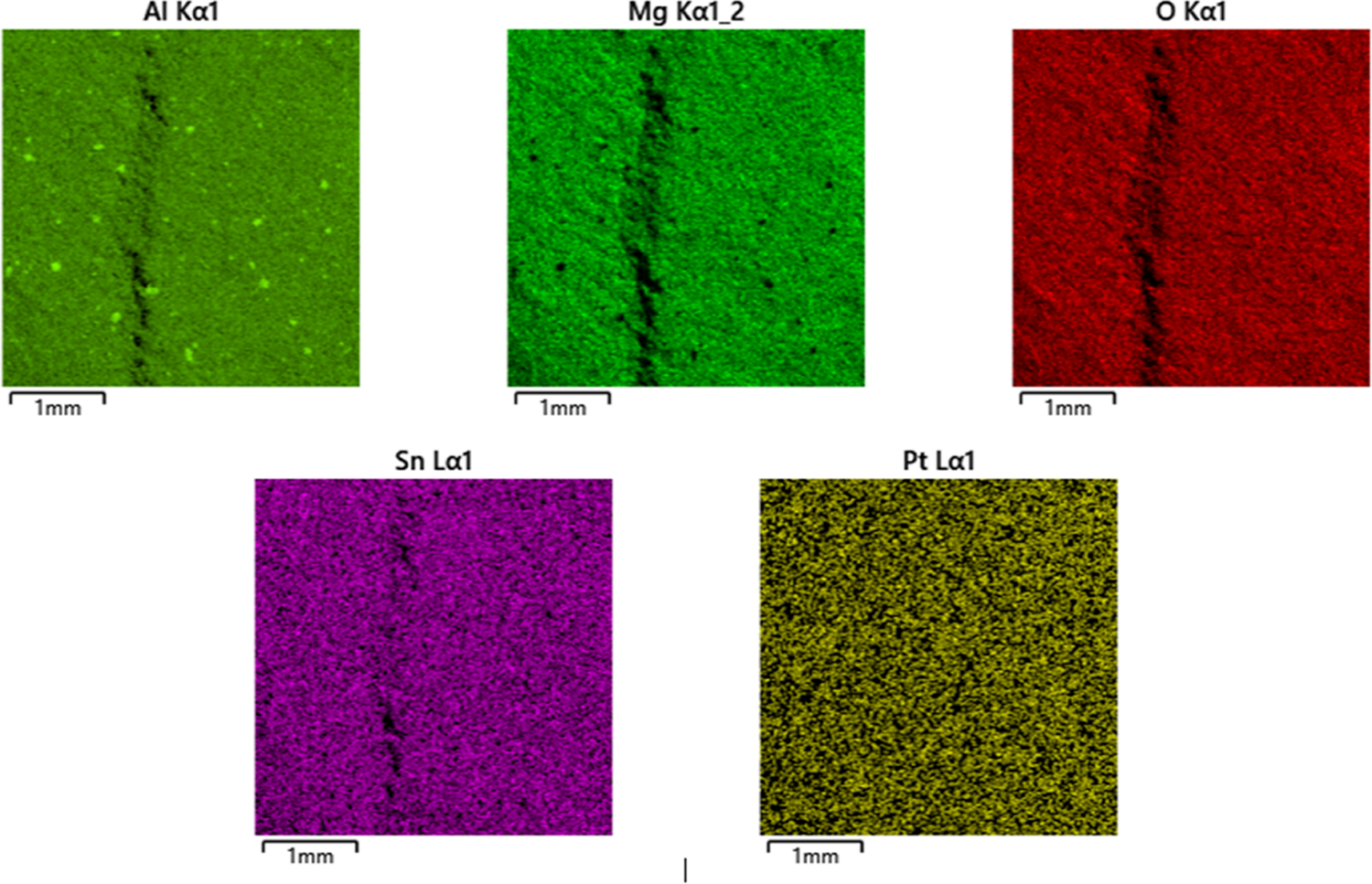
SEM–EDX image of the Sn–Pt/HTC MG70 catalyst in the pellet.

The uncalcined powdered hydrotalcite exhibits a characteristic XRD pattern.^15^ After calcination, relatively intense diffractions of MgO and diffractions related to the presence of spinels MgAl_2_O_4_^23^ are found, especially in the case of MG70 hydrotalcite pellets. Furthermore, the large peaks found in the MG70 pellets are indicative of small crystalline particles or a partially amorphous phase.^24^ Furthermore, no characteristic peaks of the active phases Pt or Sn were detected, probably due either to the high dispersion on the support or to the small size of the metal particles.^25^ The SEM–EDX image of the Sn–Pt/HTC MG70 catalyst in pellet shape is reported in Figure 2.

As evident, a good dispersion of the active species on the support has been realized with the preparation procedure.

**Figure 3:**
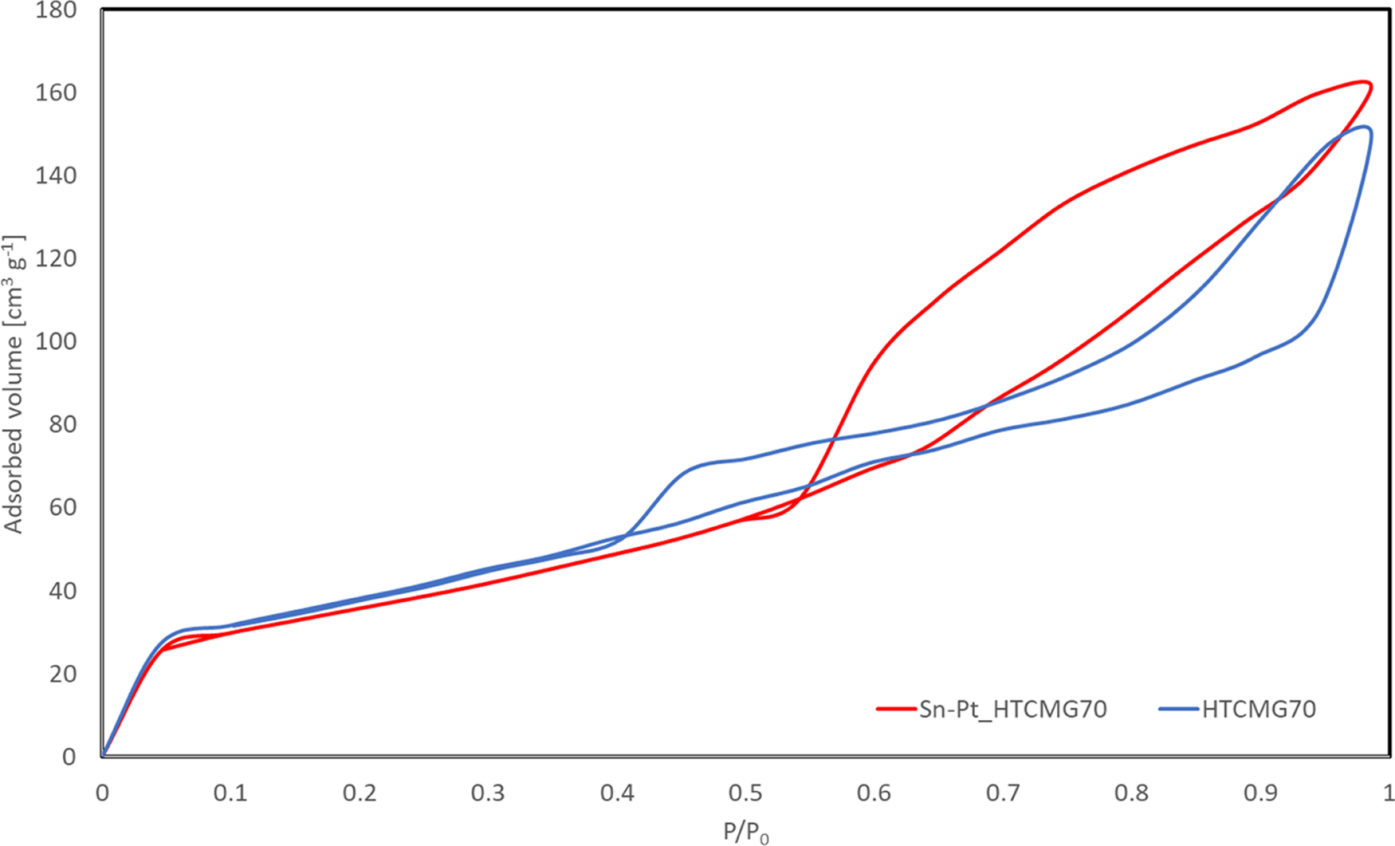
N_2_ at 77 K adsorption–desorption isotherm curves for the HTC MG70 and Sn–Pt/HTC MG70 samples in pellet shape.

The N_2_ adsorption–desorption curves are reported in Figure 3 for the MG70 bare support and the final catalyst.

The reported isotherms are both IV type according to the IUPAC nomenclature (typical of mesoporous materials), and some differences among them are evident by observing the two different hysteresis. In fact, the red one relevant to the catalytic sample is characterized by a larger hysteresis, indicating that the deposition of the active species resulted in the increase of the mesoporous features. In fact, the data reported in Table 5 confirmed the increase of both mesopore volumes and average pore radius of the catalytic sample.

**Table 5:** BET Values and Porosimetric Characteristics of Bare HTC MG70 Pellets and the Different Catalytic Samples

sample	SSA (BET)^a^, m^2^ g^–1^	mesopore volume^a^, cm^3^ g^–1^	average pore radius^a^, nm	pore area^b^, m^2^ g^–1^	pore volume^b^, cm^3^ g^–1^
HTC MG70 (pellet)	141.00	0.21	1.72	47.85	0.52
Sn–Pt/HTC MG70 (pellet)	131.00	0.23	3.17	48.84	0.37
Sn–Pt/γ-Al_2_O_3_ (powder)	140.00	0.39	4.80		
Sn–Pt/hydrotalcite (powder)	60.00	0.09	3.47		

a Evaluated with nitrogen adsorption–desorption isotherms at 77 K.

b Evaluated with mercury intrusion porosimetry.

The comparison among the SSA values of the catalysts showed a similar specific surface area in the case of Sn–Pt/γ-Al_2_O_3_ and Sn–Pt/HTC MG70, while a much lower value was found in the case of Sn–Pt/hydrotalcite powder. The effect of the addition of the active species resulted in a decrease of the surface area, as demonstrated comparing the values of HTC MG70 and Sn–Pt/HTC MG70 (Table 3); however, the catalytic sample still maintained a value higher than 100 m^2^/g. The Hg intrusion technique evidenced that the active species deposition on the HTC MG70 resulted in the decrease of the overall pore volume and in the increase of the pore area with respect to the bare pellet.

### 3.2 PDH Catalytic Activity

The results of all of the experimental tests here shown have a 95% confidence level.

**Figure 4:**
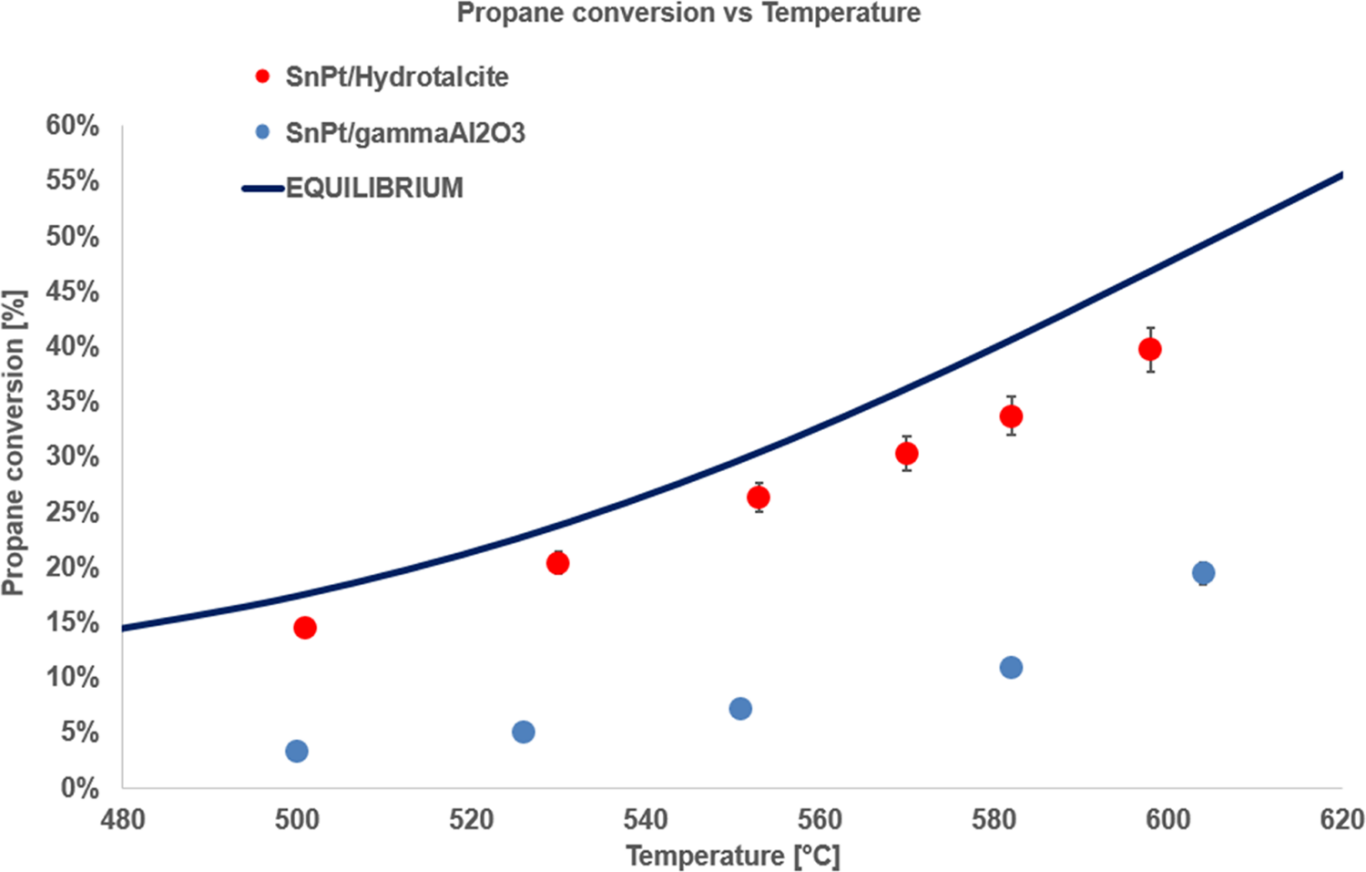
Propane conversion vs temperature for the powder samples, *T* range 500–610 °C, *P* = 1 bar, feed 80% C_3_H_8_–20% H_2_O, WHSV = 8 h^–1^.

**Figure 5:**
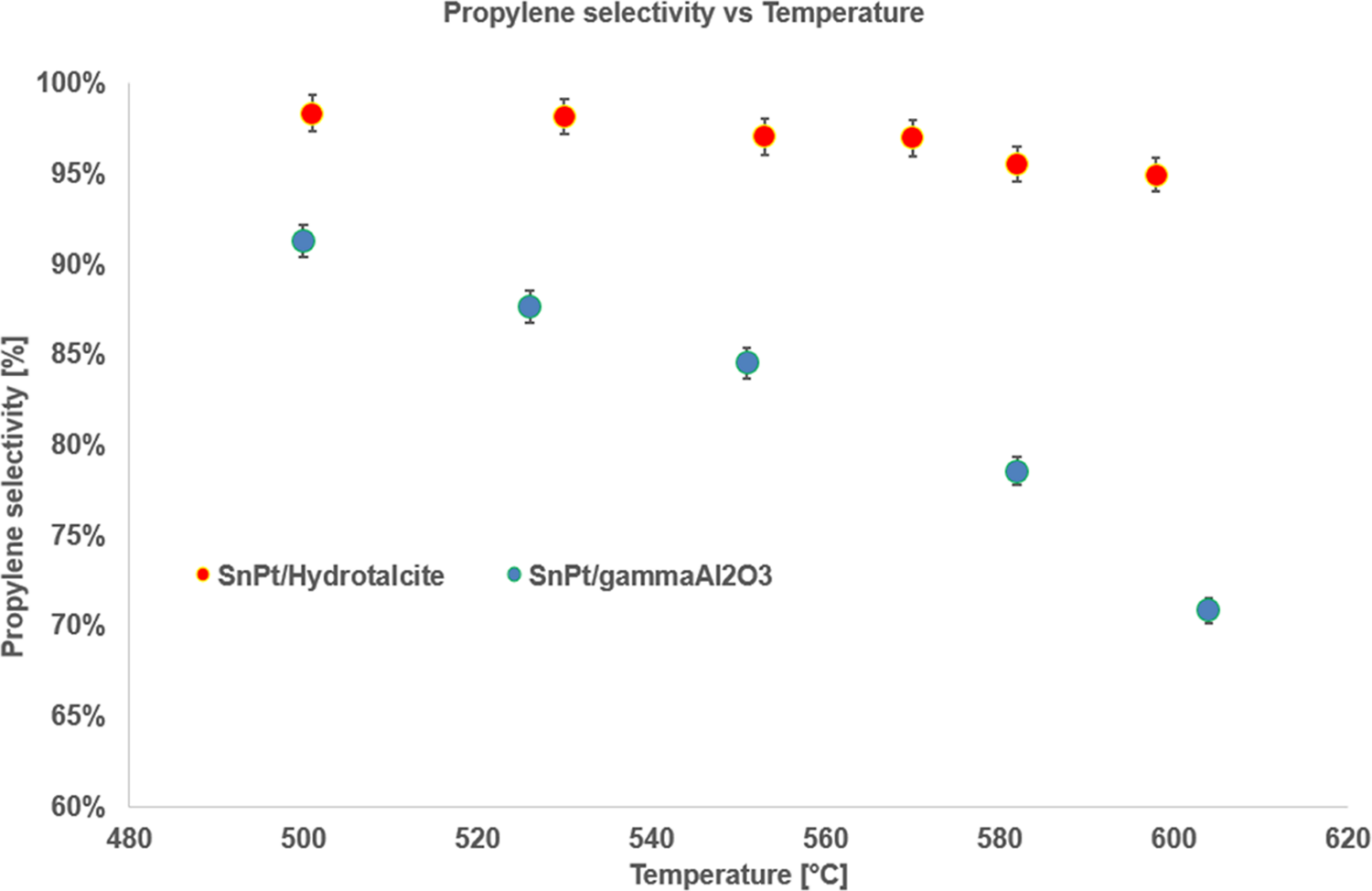
Propylene selectivity vs temperature for the powder samples, *T* range 500–610 °C, *P* = 1 bar, feed 80% C_3_H_8_–20% H_2_O, WHSV = 8 h^–1^.

Figures 4 and 5 show the catalytic activity comparisons of the powder catalysts based on γ-alumina and hydrotalcite in terms of propane conversion and propylene selectivity.

The results show the clear superiority of the hydrotalcite-based catalyst in the PDH reaction compared with the γ-alumina-based one. In particular, the former catalyst shows excellent performance both in terms of propane conversion, with values close to the thermodynamic equilibrium, and in terms of propylene selectivity, with values above 95% in the whole investigated temperature range. The alumina-based catalyst, instead, shows significantly lower performance and exceeds 90% propylene selectivity only for temperatures lower than 500 °C.

The superior performances of the hydrotalcite-based catalyst are mainly due to its better active phase dispersion compared to the γ-alumina-based catalyst, as shown by CO pulse tests, and to the basicity of the mixed oxide Mg(Al)O active sites, also highlighted by CO_2_-TPD tests, reported in Table 6.

**Table 6:** Results of CO_2_-TPD Tests

sample	basicity, μmol CO_2_ ads g_cat_^–1^
Sn–Pt/γ-Al_2_O_3_ (powder)	38
Sn–Pt/Hydrotalcite (powder)	180

**Figure 6:**
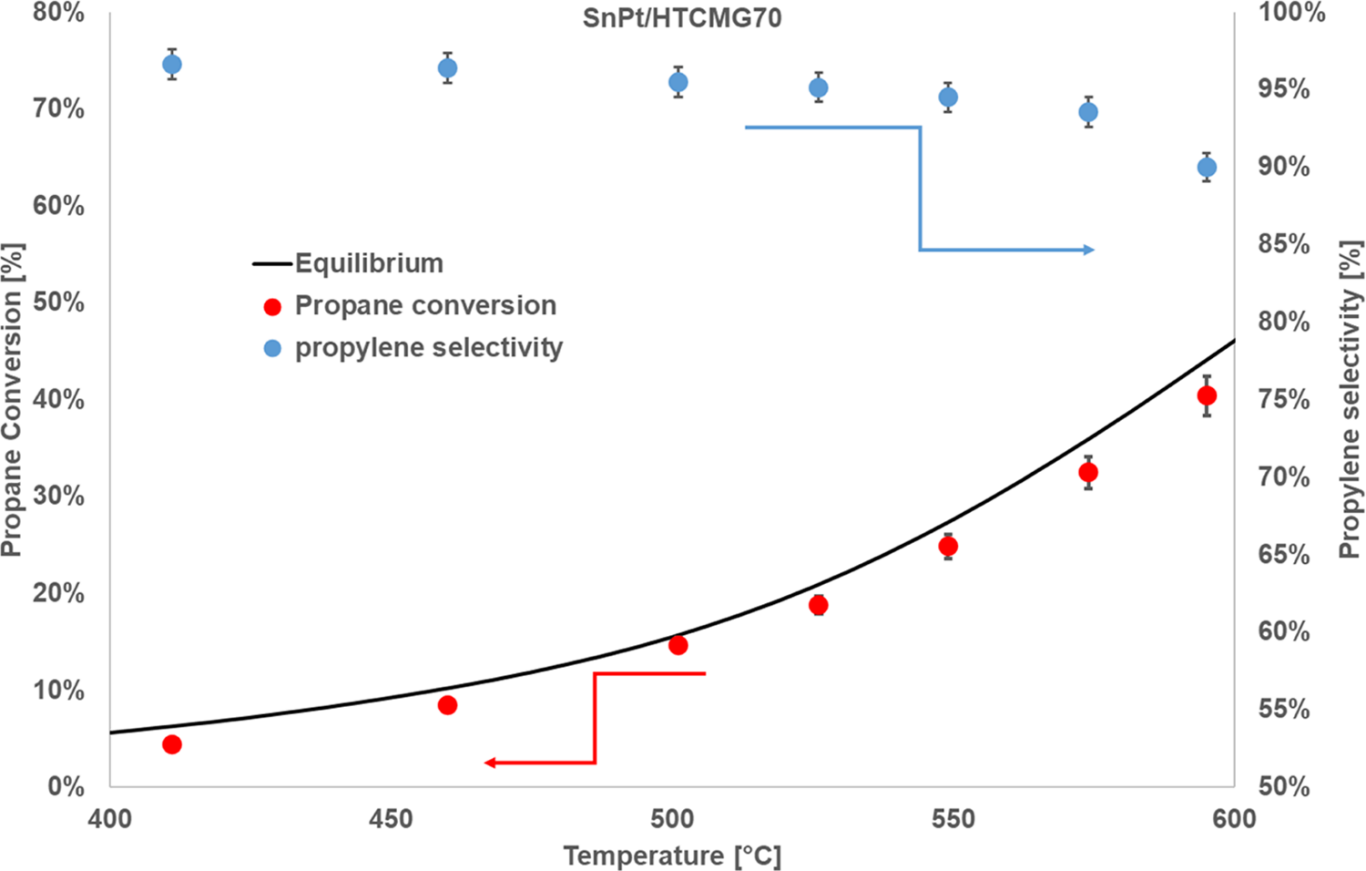
Propane conversion and propylene selectivity vs temperature for the pellet samples, *T* range 500–610 °C, *P* = 1 bar, feed 80% C_3_H_8_–20% H_2_O, WHSV = 4 h^–1^.

The excellent results shown by the hydrotalcite support led us to investigate its possible industrial applicability. For this purpose, activity and stability tests were carried out on a pellet form catalyst prepared starting from a commercial support: hydrotalcite PURALOX MG70 (by SASOL). The activity of this catalyst (SnPt/HTC MG70) was initially evaluated under atmospheric pressure at a WHSV of 4 h^–1^, and the results are shown in Figure 6 in terms of propane conversion and propylene selectivity.

This catalyst shows excellent performance for the PDH reaction under these conditions, with propane conversion values close to the thermodynamic equilibrium values and propylene selectivity higher than 90% in the whole investigated temperature range and higher than 95% up to 550 °C.

**Figure 7:**
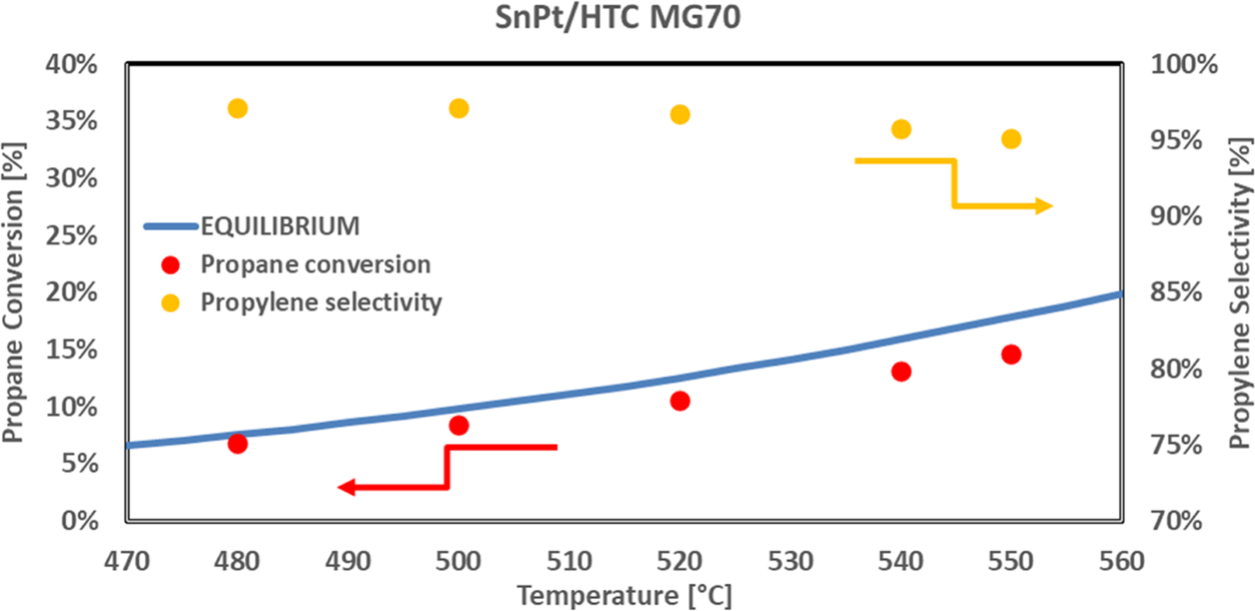
Propane conversion and propylene selectivity vs temperature for the SnPt/HTC MG70 catalyst in pellets, *T* range 480–550 °C, *P* = 5 bar, feed 80% C_3_H_8_–20% H_2_O, WHSV = 4 h^–1^.

At this point, the catalyst was tested under an operating pressure of 5 bar in order to verify its hypothetical future applicability in an integrated membrane reactor. The results of the activity tests are shown in Figure 7.

Even in these conditions, the catalyst provides excellent performance, both in terms of propane conversion, with values very close to the thermodynamic equilibrium, and in terms of propylene selectivity, with values higher than 95% in the whole investigated temperature range.

**Figure 8:**
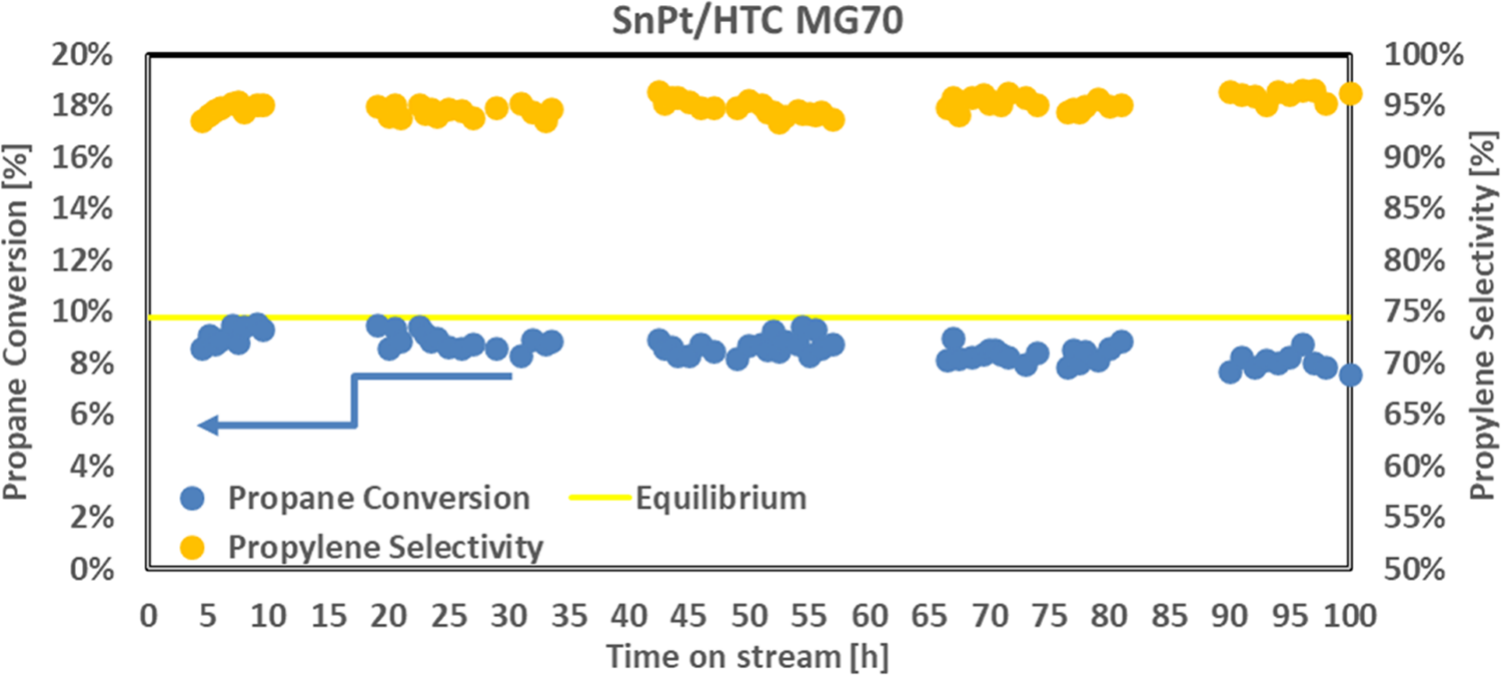
Propane conversion and propylene selectivity vs time on stream for the SnPt/HTC MG70 catalyst in pellets, *T* = 500 °C, *P* = 5 bar, feed 80% C_3_H_8_–20% H_2_O, WHSV = 4 h^–1^.

Finally, Figure 8 shows the results of a 100 h time on stream test performed under a pressure of 5 bar at a fixed temperature of 500 °C and at a WHSV of 4 h^–1^.

This catalyst shows excellent stability under these conditions, with an activity loss after 100 h of time on stream of only about 12% compared to the initial propane conversion value and with a propylene selectivity constantly higher than 95% and a slight increasing trend over time. These very important results in terms of both propane conversion and propylene selectivity during long run tests placed the developed catalyst among the most promising one if compared with some other Sn–Pt-based ones in the literature.^18,26,27^

### 3.3 Modeling Results

In the first approach, no deactivation mechanism has been included in the PDH reaction system equation set in order to obtain suitable preliminary parameter values (Table 1). Second, to investigate coke formation kinetics and gradual deactivation of the catalyst over time, deactivation mechanism expressions have been implemented, as detailed in the following lines.

The least-squares method was used to optimize the rate parameters in the developed model by comparing experimental data with model predictions. The kinetic parameters were determined with a 95% confidence level.

The kinetic parameter values and related objective functions OF values, obtained for the propane dehydrogenation reaction system, at different WHSVs are presented in Tables S1 and S2, respectively.

The further optimization of the obtained data, mandatory for making the model suitable for all of the investigated operating conditions, is reported in Table S3.

The data reported in Table S3 evidenced a good agreement of the activation energies of the proposed reactions with the literature.^18^

**Figure 9:**
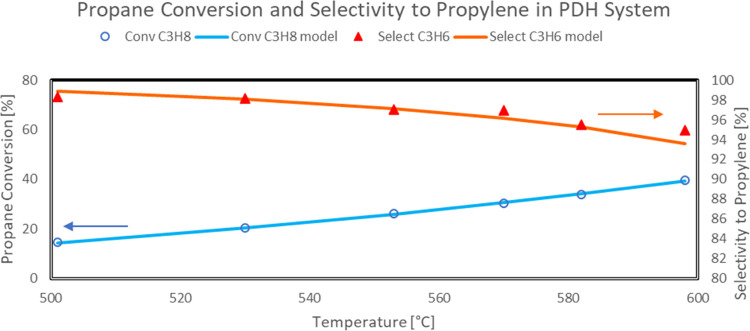
Approach of predicted model values to experimental propane conversion and selectivity to propylene in a PDH reaction system (Sn–Pt/HTC MG70, WHSV = 4 h^–1^, C_3_H_8_/H_2_O = 80:20, *P* = 5 bar).

As an example, the comparison between experimental propane conversion (open symbol) and propylene selectivity (solid symbols) and model predicted values (solid line) as a function of reaction temperature in the PDH test performed at 4 h^–1^ is reported in Figure 9.

**Figure 10:**
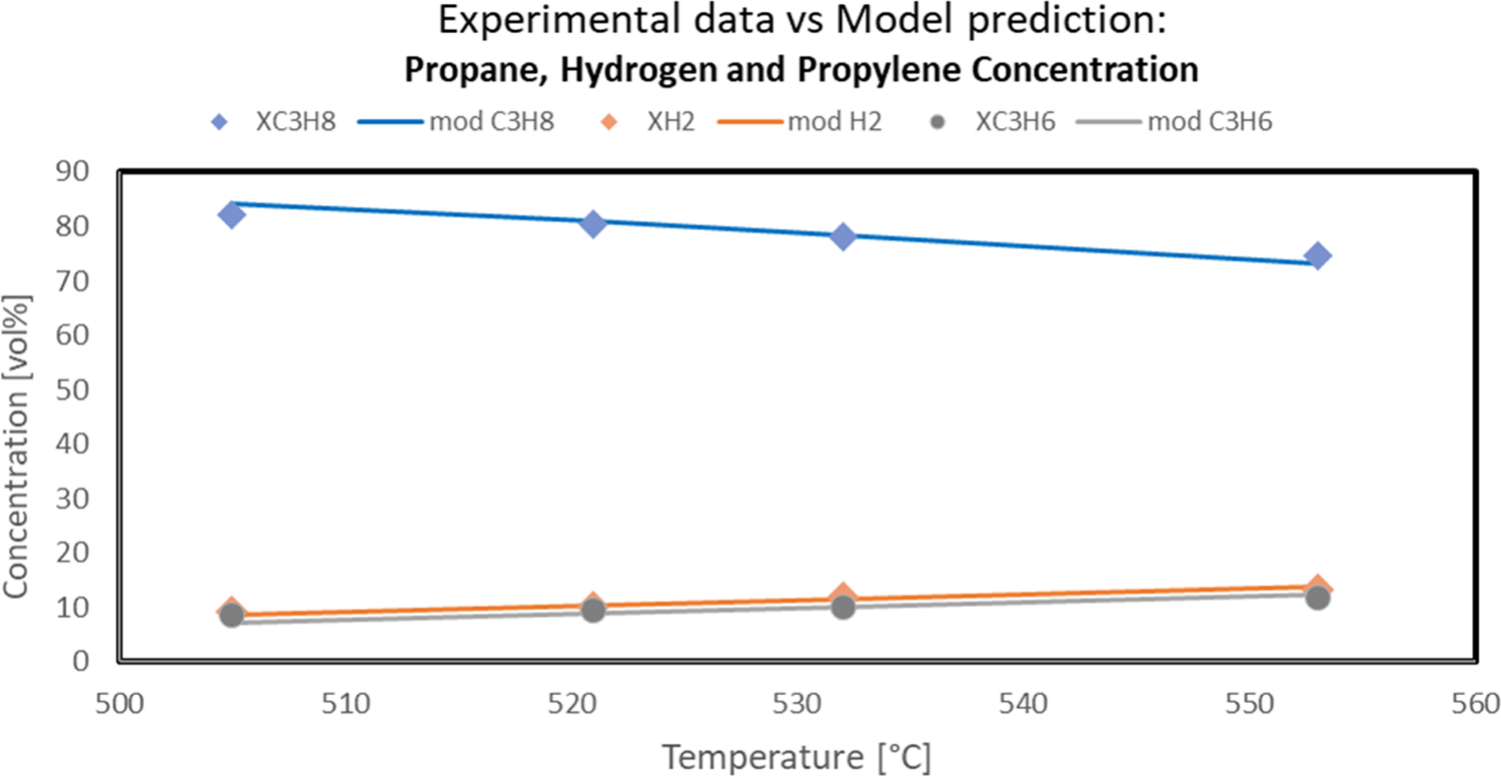
Approach of predicted model values to experimental outlet propane, hydrogen, and propylene concentration in the PDH reaction system (Sn–Pt/HTC MG70, WHSV = 4 h^–1^, C_3_H_8_/H_2_O = 80:20, *P* = 5 bar).

**Figure 11:**
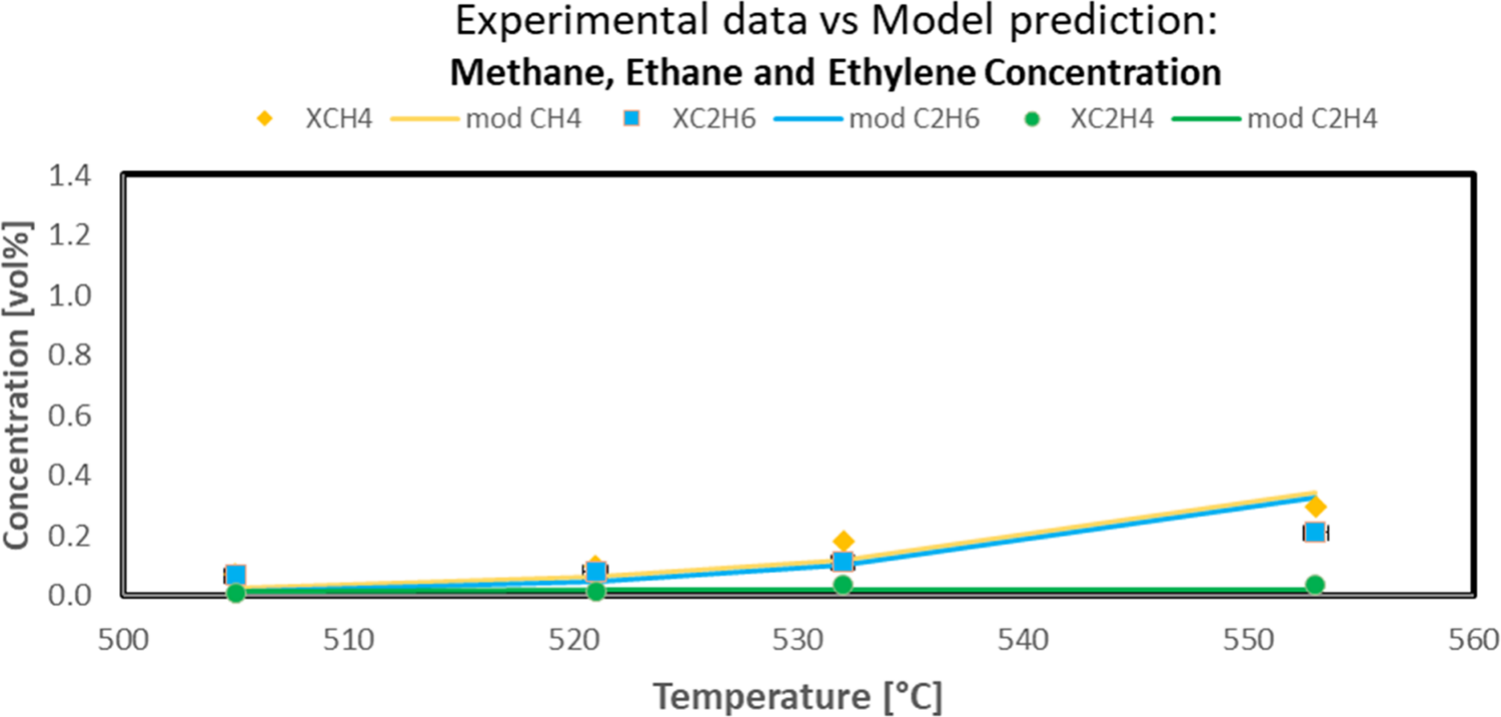
Approach of predicted model values to experimental outlet methane, ethane, and ethylene concentration in the PDH reaction system (Sn–Pt/HTC MG70, WHSV = 4 h^–1^, C_3_H_8_/H_2_O = 80:20, *P* = 5 bar).

More in detail, the capability of the developed model to fit the experimental data also in terms of gas composition is reported in the following figures. In particular, the comparison between experimental propane, hydrogen, propylene, methane, ethane, and ethylene concentrations (symbols) and model predicted values (solid line) as a function of reaction temperature in the PDH reaction system is reported in Figures 10 and 11.

The data shown in the above-reported figures highlighted the very good agreement between the experimental and the model data, thus confirming the validity of the assumptions made for the model development.

Once the kinetic parameters have been calculated, the suitability of the models to represent the kinetic data can be assessed. The stability test used for the model optimization and validation is the one previously shown (Figure 8).

**Figure 12:**
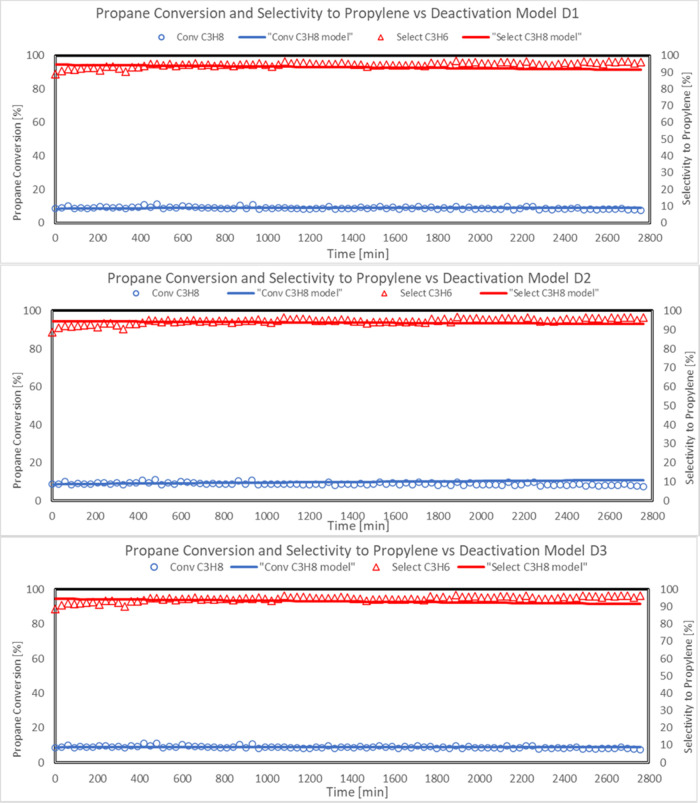
Approach of predicted model values for deactivation models D1, D2, and D3 to experimental propane conversion and selectivity to propylene in the PDH reaction system during stability test (Sn–Pt/HTC MG70, WHSV = 4 h^–1^, C_3_H_8_/H_2_O = 80:20, *T* = 500 °C, *P* = 5 bar).

Comparison between experimental propane conversion (open symbol) and propylene selectivity (solid symbols) and model predicted values (solid line) as a function of reaction time during stability test in the PDH reaction system are reported in Figure 12 for the different proposed deactivation models (Table 4).

**Figure 13:**
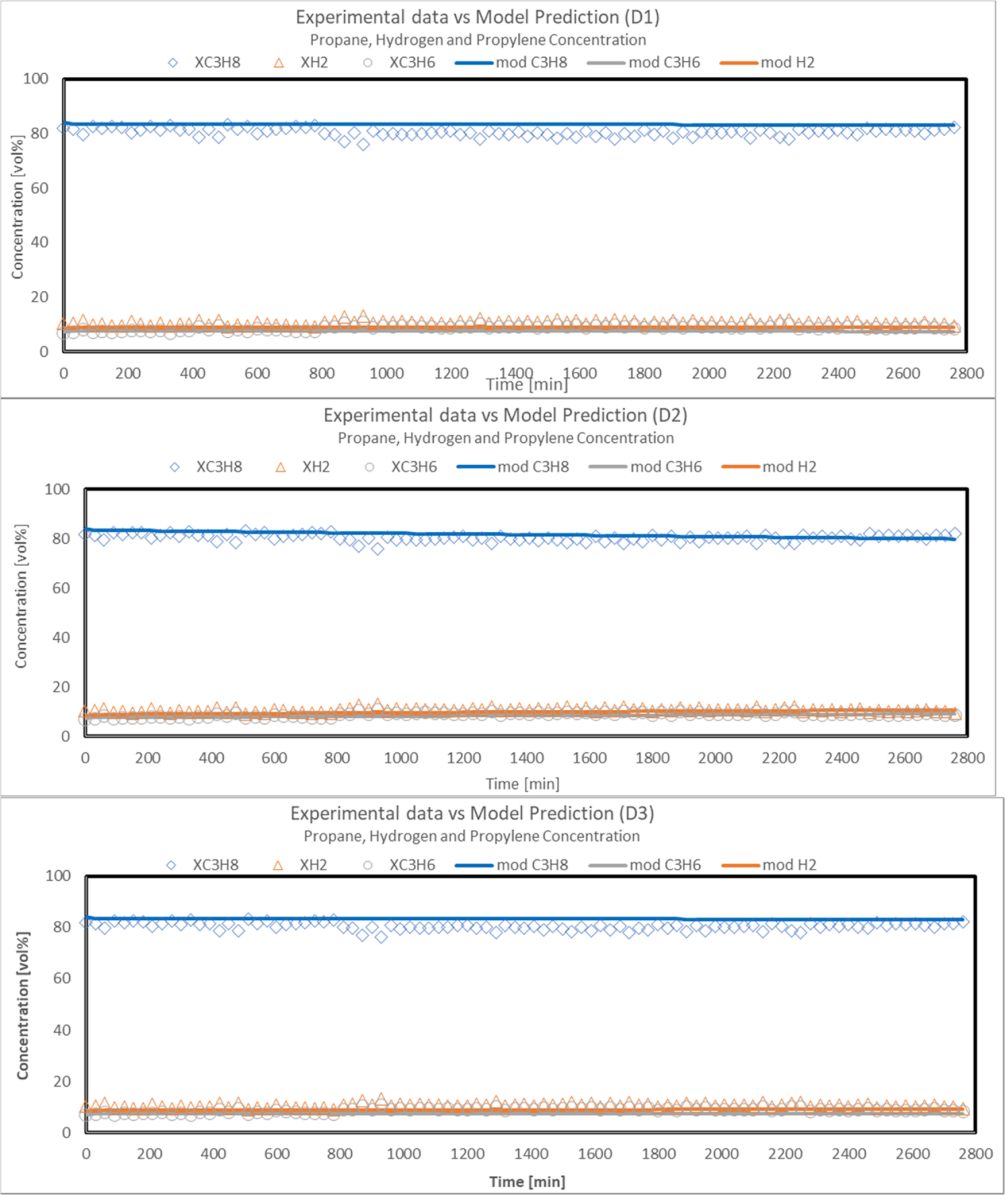
Approach of predicted model values for deactivation models D1, D2, and D3 to experimental propane, hydrogen, and propylene concentration in the PDH reaction system during stability test (Sn–Pt/HTC MG70, WHSV = 4 h^–1^, C_3_H_8_/H_2_O = 80:20, *T* = 500 °C, *P* = 5 bar).

More in detail, the capability of the developed model to fit the experimental data also in terms of gas composition is reported in the following figures. In particular, the comparison between experimental propane, hydrogen, propylene, concentrations (symbols), and model predicted values (solid line) as a function of time on stream in the PDH reaction system is reported in Figure 13 for deactivation models D1, D2, and D3.

Predicted values of propane conversion and propylene selectivity were in good agreement with the experimental data.

Calculated values of the OF function, obtained from the comparison between predicted deactivation modes D1, D2, and D3 and stability test experimental data, are reported in Table S4.

Deactivation model D2 presents the best fitting of experimental data with a lower value of the OF (Table S4). Therefore, the overall kinetic parameters are listed in Table 7.

**Table 7:** Kinetic Parameter Values (with Confidential Intervals) for the PDH Rection Set (Comprising Deactivation Mechanism D2) for Sn–Pt/HTC MG70

reaction	kinetic parameter	
	parameter	value
Propane Dehydrogenation		
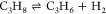	*k*_01_	0.00003 mmol/(g·min·bar)
	*E*_a1_	72.230 ± 1.62 kJ/mol
	*K*_0_	4427 ± 98.99
	Δ*H*	–79.998 ± 1.7888 kJ/mol
	γ_01_	9.98 × 10^13^ ± 2.23 × 10^12^ g_cat_/g_coke_
	*E*_aγ1_	78.353 ± 1.75 kJ/mol
	γ_2_	2.00 × 10^14^ ± 4.47 × 10^12^ g_cat_/g_coke_
	γ_3_	0
Propane Cracking		
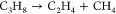	*k*_02_	1.3 × 10^–4^ ± 2.91 × 10^–6^ mmol/(g·min·bar)
	*E*_a2_	381.865 ± 6.75 kJ/mol
Ethylene Hydrogenation		
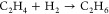	*k*_03_	140.00 ± 3.13 mmol/(g·min·bar^2^)
	*E*_a3_	150.00 ± 3.35 kJ/mol
Coke Formation		
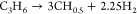	*k*_01C_	5.68 × 10^–12^ ± 1.27 × 10^–13^ mg_cat_/(mg_coke_·min)
	*E*_a1C_	41.325 ± 0.92 kJ/mol
	*k*_02C_	2.01 × 10^–5^ ± 4.49 × 10^–7^ mg_coke_/(mg_cat_·min)
	*E*_a2C_	73.567 ± 1.64 kJ/mol
	*C*_max_	0.000494 ± 1.1 × 10^–5^ mg_coke_/mg_cat_

### 3.4 Characterization of the Spent Catalytic Samples

**Figure 14:**
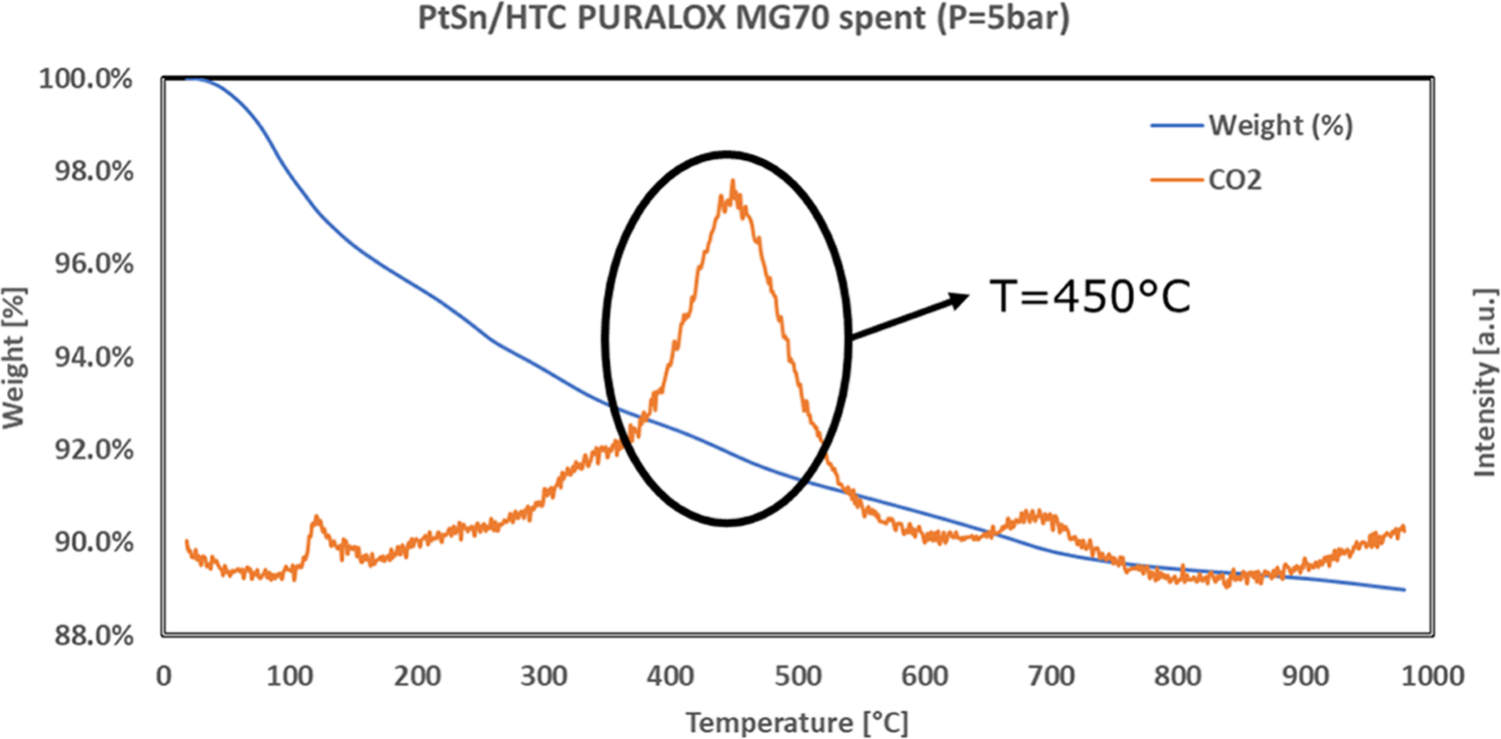
TG analysis results for the spent Pt–Sn-based catalyst prepared starting from HTC MG70.

The characterization of the spent catalyst was focused on the characterization of the coke formed during the time on stream test in order to optimize a potential regeneration procedure. The results of the TG analysis are reported in Figure 14.

From Figure 14, it can be seen that the HTC MG70-based catalyst shows a weight loss due to the coke combustion (confirmed by the increase in the CO_2_ signal detected at the output of the system) in the temperature range of 370–580 °C, with a CO_2_ production peak detected at about 450 °C. This result shows how the HTC MG70-based catalyst could be regenerated at a relatively low temperature. The carbon formation rate was calculated using the following equation
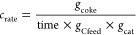
10

As a result of this calculation, a carbon formation rate of 5.4 × 10^–8^ has been obtained, which is also consistent with the one obtained by the developed model.

**Figure 15:**
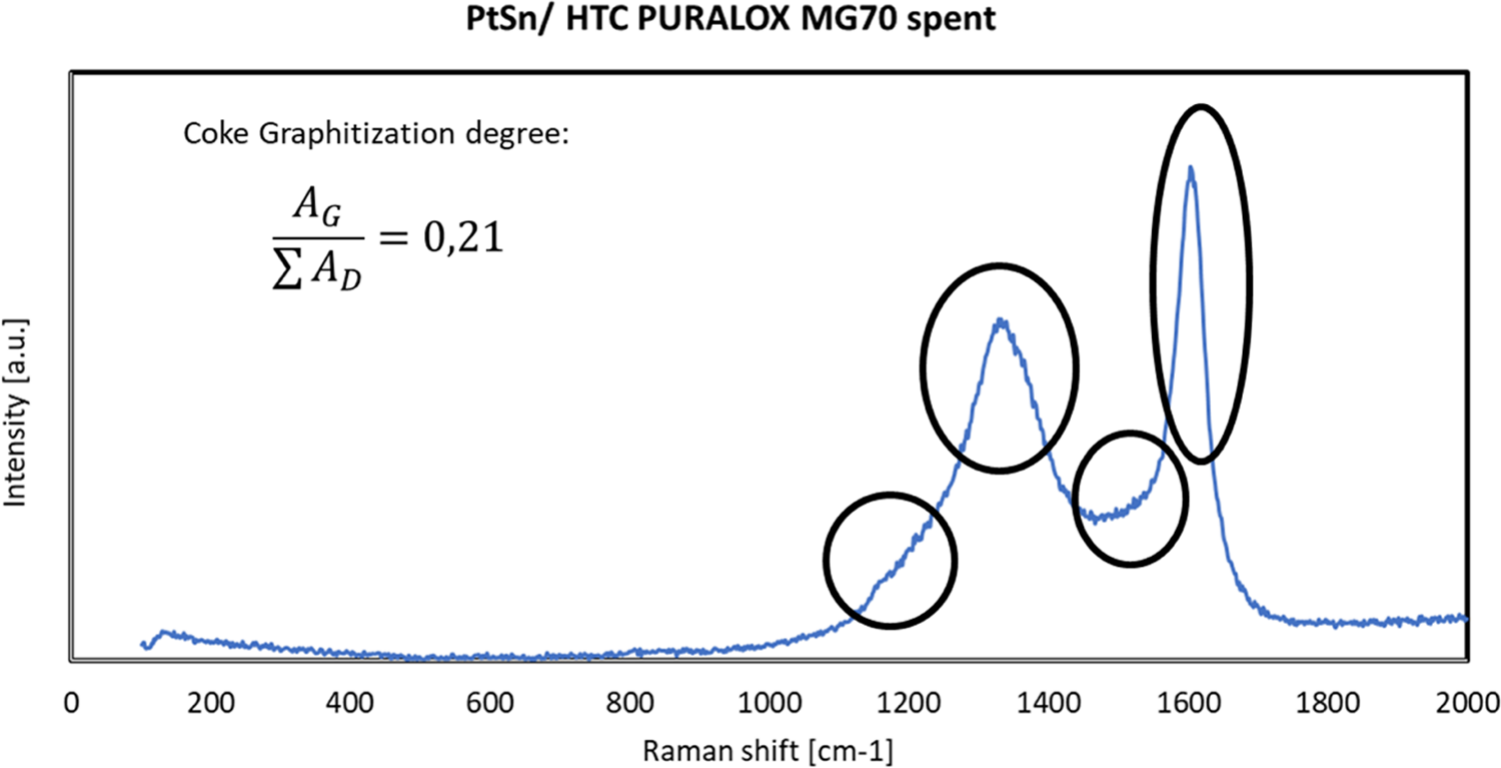
Raman spectrum of the Pt–Sn/HTC MG70 spent catalyst.

The Raman spectrum of the spent catalyst is shown in Figure 15.

The above-reported figure evidenced that the classical peaks relevant to carbonaceous materials are present:^28^

- R.S. = 1600 cm^–1^—ideal graphitic lattice vibration (G band);
- R.S. = 1350 cm^–1^—in-plane defects and heteroatoms (D1 band);
- R.S. = 1500 cm^–1^—amorphous carbon (D3 band);
- R.S. = 1150 cm^–1^—disordered graphitic lattice (D4 band).

**Figure 16:**
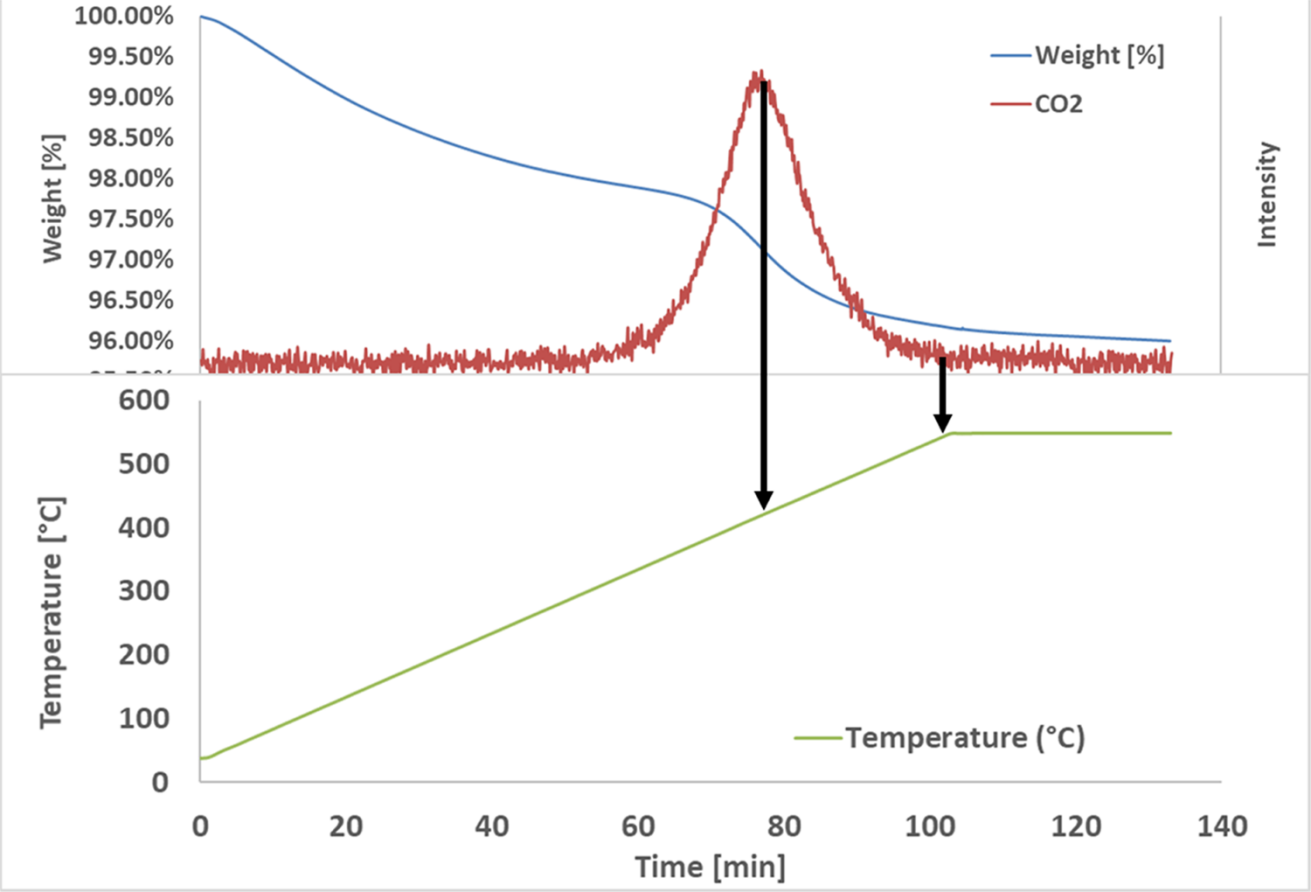
Preliminary regeneration of a sample took from the spent catalyst: from R.T. to 550 °C (ramp 5 °C/min) and isothermal at 550 °C for 30 min in an air flow.

The calculation of the graphitization degree as the ratio between the area of peak G and the area of all of the D peaks highlighted that a low value is obtained, a sign of the formation of a more disordered coke, which could be oxidized at lower temperature than a more ordered and graphitized one. This spectrum confirms the result of the TG analysis, in which coke combustion occurs at a temperature lower than the classical coke combustion temperature (about 600 °C). After that, a preliminary regeneration of the spent catalyst was performed by means of the TG analysis under the following operating conditions: from R.T. to 550 °C (ramp 5 °C/min) and isothermal at 550 °C for 30 min in an air flow. The results are shown in Figure 16.

**Figure 17:**
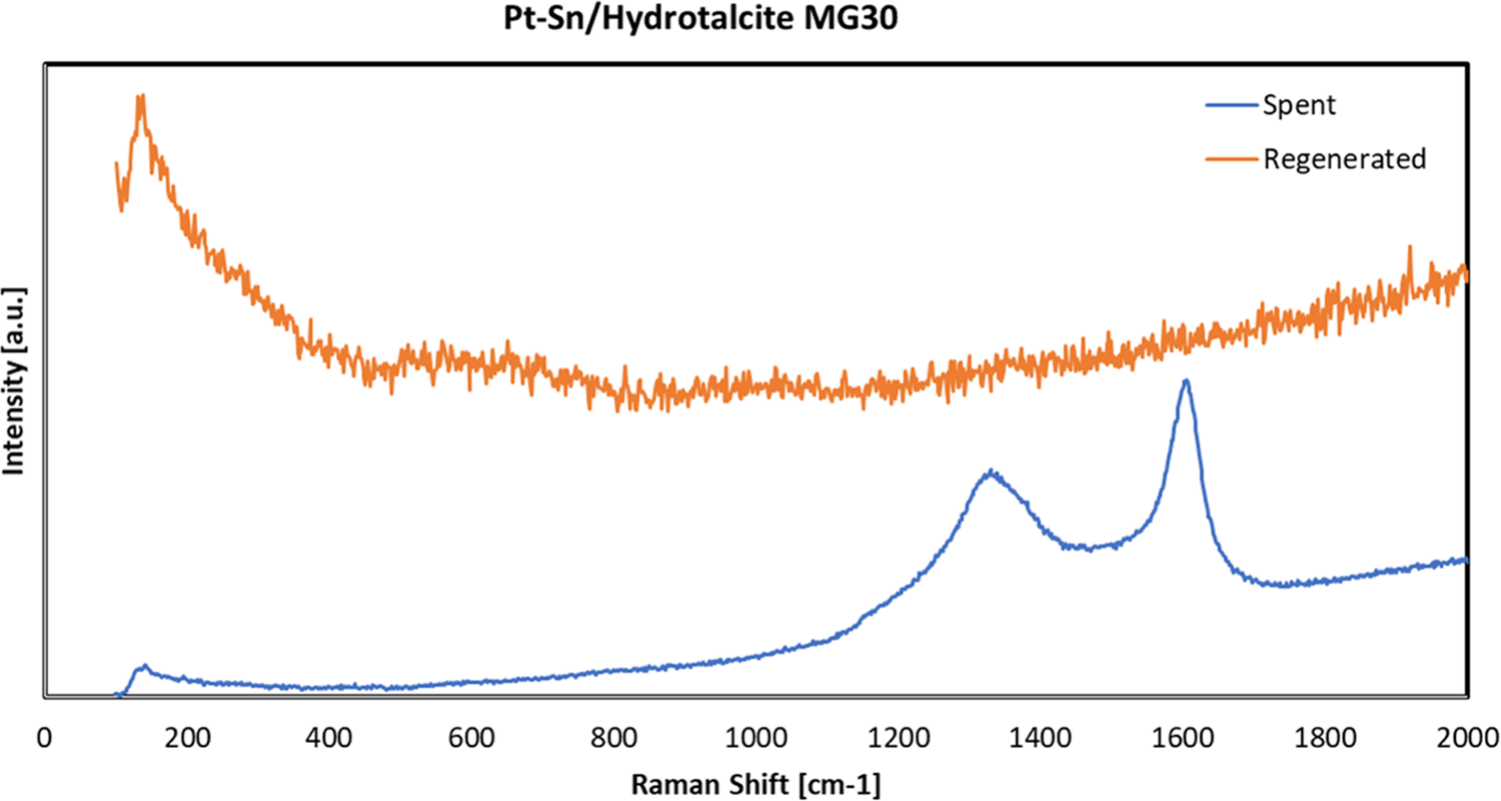
Raman spectra of the spent (blue curve) and regenerated (orange curve) catalyst samples.

As evident, the spent catalyst is fully regenerated by using this procedure: in fact, CO_2_ is emitted from coke combustion up to 500 °C, and after that, no more CO_2_ is present. The full regeneration of the spent catalyst is also confirmed by Raman analysis (Figure 17), in which the peaks relevant to coke are not present after regeneration (orange curve).

### 3.5 Cyclic Activity Tests

After the very interesting results of the TG tests, our efforts were devoted to the optimization of the regeneration procedure for the pellet catalyst and for performing cyclic stability/regeneration tests in order to verify the behavior of the catalyst after the regeneration step. Regarding the regeneration procedure, the following steps were followed:

(1) Reaction at 550 °C;
(2) Cooling up to 500 °C in a nitrogen flow (≈20 min);
(3) Regeneration for coke burning at 500 °C with a gradual increase in O_2_ concentration (from 2 up to 20%) in a N_2_ flow (≈3.5 h);
(4) Heating up to 600 °C in 5 vol % H_2_ in a N_2_ flow (≈20 min);
(5) Reduction in 5 vol % H_2_ in a N_2_ flow at 600 °C (1 h).

A gradual O_2_ increase (point 3) was adopted in order to avoid uncontrolled temperature increases in the catalytic bed with consequent active metal sintering corresponding to worst selectivity. It is also essential to avoid overheating to 600 °C in order to preserve the catalyst performance and integrity.

The fresh and regenerated Sn–Pt/HTC MG70 pellet catalysts have also been characterized by means of CO_2_-TPD in order to verify if the regeneration procedure could affect its basicity, and the results are reported in Table 8.

**Table 8:** Results of CO_2_-TPD and CO Pulse Tests for the Fresh and Regenerated Sn–Pt/HTC MG70 Catalysts in Pellets

sample	basicity, μmol CO_2_ ads g_cat_^–1^	dispersion, %
Sn–Pt/HTC MG70 fresh	207	62.7
Sn–Pt/HTC MG70 regenerated	200	61

The results reported in Table 8 evidenced how the adopted regeneration procedure did not seriously affect the basicity of the catalyst, which in principle could have the same catalytic performance as the fresh one. Moreover, the CO pulse test highlighted that the Pt dispersion in the regenerated catalyst (after the stability test) was slightly different from the fresh one. This last result may be ascribed to a very low grade of Pt sintering. The chemical composition of the fresh and spent catalysts is reported in Table 9.

**Table 9:** Chemical Composition of the Fresh and Regenerated Sn–Pt/HTC MG70 Catalysts in Pellets

sample	Pt, wt %	Sn, wt %	Al, wt %	Mg, wt %
Sn–Pt/HTC MG70 fresh	0.85	0.60	26.91	71.64
Sn–Pt/HTC MG70 regenerated	0.84	0.64	26.84	71.68

The above-reported data evidenced a quite good agreement between the evaluated and the nominal active species loading, which did not change after the reaction and regeneration steps.

**Figure 18:**
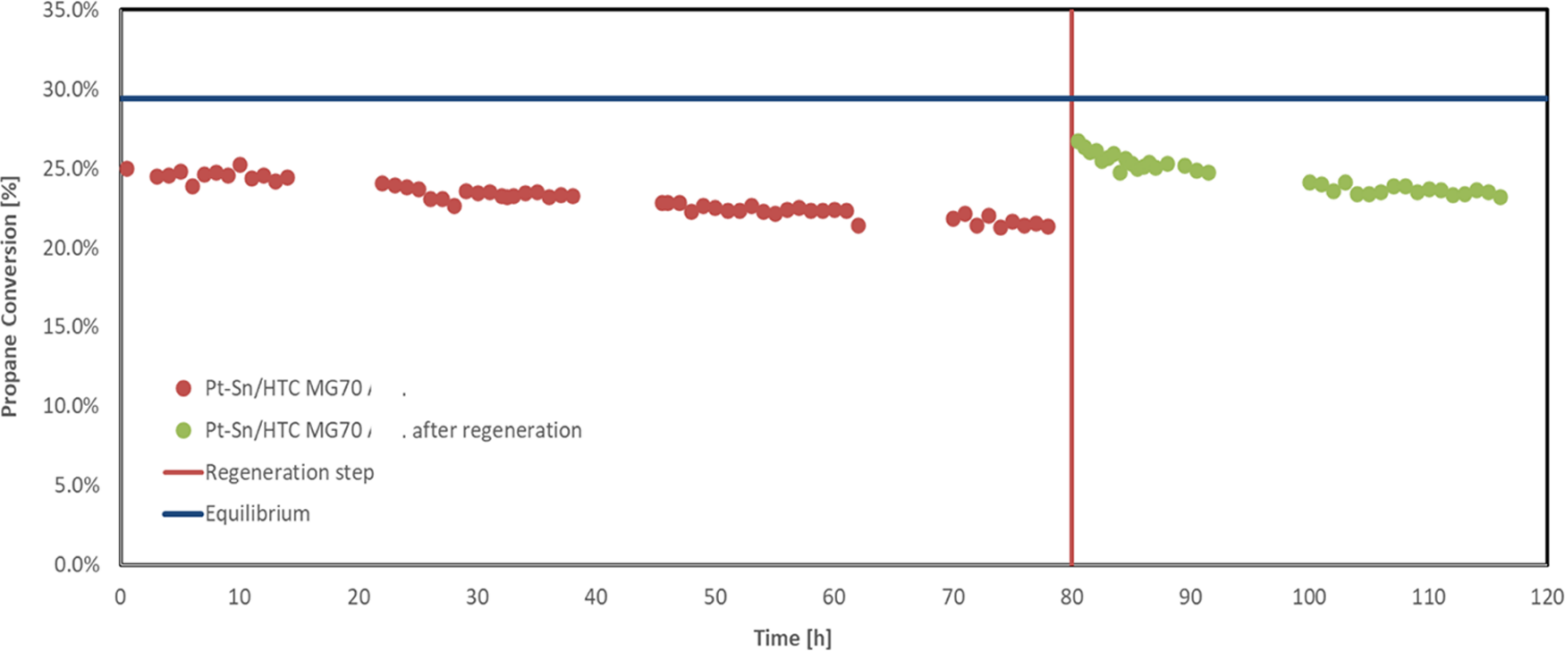
Results relevant to a cyclic test in terms of propane conversion vs time on stream for the Sn–Pt/HTC MG70 catalyst in pellets, *T* = 550 °C, *P* = 1 bar, feed 80% C_3_H_8_–20% H_2_O, WHSV = 4 h^–1^.

**Figure 19:**
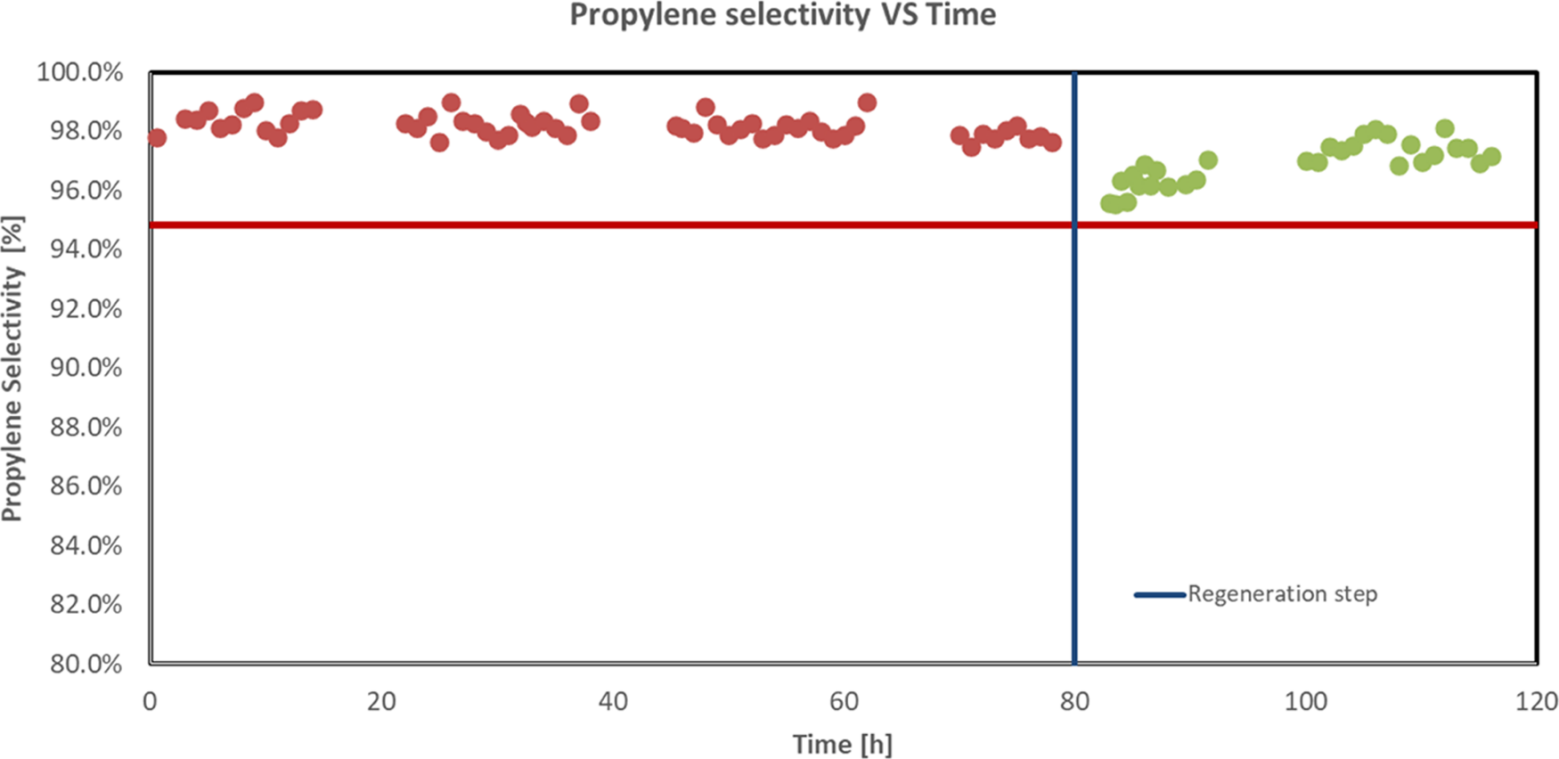
Results relevant to a cyclic test in terms of propylene selectivity vs time on stream for the Sn–Pt/HTC MG70 catalyst in pellets, *T* = 550 °C, *P* = 1 bar, feed 80% C_3_H_8_–20% H_2_O, WHSV = 4 h^–1^.

The results of a cyclic test performed at 1 bar and 550 °C are reported in Figures 18 and 19 in terms of propane conversion and propylene selectivity vs *T*, respectively.

The above-reported results highlighted the positive effect of the regeneration procedure: the catalyst recovers the initial catalytic activity in terms of both propane conversion and propylene selectivity, and the decrease rate of the former is the same as before the regeneration. This last result is noteworthy since it evidenced how the very low sintering of Pt did not affect the catalytic performance of the catalyst. These very interesting results seem to indicate the possibility to use this catalyst for industrial purposes. In particular, the very low coke tendency evidenced by the activity tests (only a decrease from 25 up to 22% in the propane conversion, corresponding to about 12% with respect to the initial value, was observed, with no decrease in the selectivity), the high selectivity to propylene (>95%), and the easy regenerability of the proposed catalyst make it a very good alternative to the actually used catalysts for the PDH process.

## 4 Conclusions

In this work, the performance in the PDH reaction of different Sn–Pt catalysts prepared starting by alumina- and hydrotalcite-based supports is investigated in terms of propane conversion and selectivity to propylene. The experimental tests evidenced that the best performance was obtained by using the catalyst prepared on commercial pellets of hydrotalcite PURALOX MG70 by SASOL. This catalyst has shown, under pressure conditions of 1 and 5 bar (in order to evaluate the potential future application in integrated membrane reactors), propane conversion values close to the thermodynamic equilibrium ones in all of the investigated temperature ranges (500–600 °C) and the selectivity was always higher than 95%. So, this catalyst was also tested in a stability run, performed at 500 °C and 5 bar for 100 h: the results highlighted the loss of only 12% in the propane conversion, with no changes in the selectivity to propylene. Properly designed experimental tests have also been performed in order to evaluate the kinetic parameters, and the comparison between the modeling and the experimental results evidenced a very good fitting also considering the catalyst deactivation due to coking. The characterization of the coke formed on the spent catalyst gave indications for the optimization of the regeneration procedure, and cyclic stability/regeneration tests have been performed in order to verify the behavior of the catalyst after the regeneration step. The results of these tests evidenced the positive effect of the regeneration procedure: the catalyst recovers the initial catalytic activity in terms of both propane conversion and propylene selectivity, and the decrease rate of the former is the same as before the regeneration. These very interesting results seem to indicate the possibility to use this catalyst for industrial purposes.

## Supporting Information Available

The tables with the kinetic parameters calculated by means of the developed mathematical model (PDF)
