# Supplementary material for: Modeling the Selectivity of Hydrotalcite-Based Catalyst in the Propane Dehydrogenation Reaction
Source: Ind Eng Chem Res. 2023 Oct 9;62(41):16622–37. doi: 10.1021/acs.iecr.3c01076 (PMC10588453; doi:10.1021/acs.iecr.3c01076)
Supplement: Supplementary file 1 — ie3c01076_si_001.pdf [file ie3c01076_si_001.pdf]

Modelling the selectivity of hydrotalcite based catalyst in the propane dehydrogenation reaction

Giovanni Festa[1], Palma Contaldo[1], Marco Martino[1], Eugenio Meloni[1*], Vincenzo Palma[1]

[1] University of Salerno, Department of Industrial Engineering, Via Giovanni Paolo II 132, 84084 Fisciano, SA, Italy.

*Correspondence: emeloni@unisa.it;

This supporting information file contains the tables with the kinetic parameters calculated by means of the developed mathematical model.

Table S1: Kinetic parameters values (with the confidence intervals) for PDH Rection set for Sn-Pt/HTCMG70 catalyst, C_3H_8:H_2O = 80%:20%, P = 5 bar.

**Reaction**	**Kinetic parameters**						
Propane Dehydrogenation	Parameter	UOM	WHSV 4 h^{-1}	WHSV 8 h^{-1}	WHSV 12 h^{-1}	WHSV 24 h^{-1}	WHSV 36 h^{-1}
R1 $C_3H_8 \rightleftharpoons C_3H_6 + H_2$	k_{01}	mmol / (g*min*bar)	0.21893 ± 0.0049	0.38127 ± 0.0085	0.53879 ± 0.012	0.89163 ± 0.020	1.10284 ± 0.025
	E_{a1}	kJ/mol	72.230 ± 1.62	74.010 ± 1.65	72.228 ± 1.62	74.231 ± 1.66	74.031 ± 1.66
	K_0	-	4427 ± 98.99	4401 ± 98.41	4402 ± 98.43	4401 ± 98.41	4361 ± 97.51
	ΔH	kJ/mol	-79.998 ± 1.7888	-79.982 ± 1.7884	-79.992± 1.7886	-79.982 ± 1.7884	-79.982 ± 1.7884
Propane Cracking							
R2 $C_3H_8 \rightarrow C_2H_4 + CH_4$	k_{02}	mmol/(g*min*bar)	0.00212 ± 4.74 * 10^{-5}	0.00211 ± 4.72 * 10^{-5}	0.00208 ± 4.65 * 10^{-5}	0.00205 ± 4.58 * 10^{-5}	0.00205 ± 4.58 * 10^{-5}
	E_{a2}	kJ/mol	301.865 ± 6.75	301.865 ± 6.75	301.865 ± 6.75	301.865 ± 6.75	301.865 ± 6.75
Ethylene hydrogenation							
R3 $C_2H_4 + H_2 \rightarrow C_2H_6$	k_{03}	mmol/(g*min*bar^2)	140.4 ± 3.14	140.4 ± 3.14	140.4 ± 3.14	140.4 ± 3.14	140.4 ± 3.14
	E_{a3}	kJ / mol	200.00 ± 4.47	200.00 ± 4.47	200.00 ± 4.47	200.00 ± 4.47	200.00 ± 4.47
Coke formation							

R4 $C_3H_6 \rightarrow 3CH_{0.5} + 2.25\ H_2$	k_{01C}	$mg_{cat}/(mg_{coke} * min)$	$5.68 * 10^{-14} \pm 1.27 * 10^{-15}$	$5.68 * 10^{-14} \pm 1.27 * 10^{-15}$	$5.68 * 10^{-14} \pm 1.27 * 10^{-15}$	$5.68 * 10^{-14} \pm 1.27 * 10^{-15}$	$5.68 * 10^{-14} \pm 1.27 * 10^{-15}$
	E_{a1C}	kJ/mol	40.352 ± 0.902	40.352 ± 0.902	40.352 ± 0.902	40.352 ± 0.902	40.352 ± 0.902
	k_{02C}	$mg_{coke}/(mg_{cat} * min)$	$7.50 * 10^{-3} \pm 1.67 * 10^{-4}$	$7.89 * 10^{-3} \pm 1.76 * 10^{-4}$	$1.19 * 10^{-2} \pm 2.66 * 10^{-4}$	$1.37 * 10^{-2} \pm 3 * 10^{-4}$	$1.97 * 10^{-2} \pm 4.4 * 10^{-4}$
	E_{a2C}	kJ/mol	53.568 ± 1.197	53.568 ± 1.197	53.588 ± 1.198	53.588 ± 1.198	53.417 ± 1.194
	C_{max}	mg_{coke}/mg_{cat}	$0.000494 \pm 1.1 * 10^{-5}$	$0.000494 \pm 1.1 * 10^{-5}$	$0.000494 \pm 1.1 * 10^{-5}$	$0.000494 \pm 1.1 * 10^{-5}$	$0.000494 \pm 1.1 * 10^{-5}$

Table S2: Objective function (OF) values for PDH Rection set for Sn-Pt/HTCMG70 catalyst, at different WHSV.

Kinetic model for PDH reaction System for:	OF values
WHSV 4 h^{-1}	0.589
WHSV 8 h^{-1}	0.489
WHSV 12 h^{-1}	0.418
WHSV 24 h^{-1}	0.484
WHSV 36 h^{-1}	0.506

Table S3: Optimized kinetic parameters values (with the confidence intervals) for PDH Rection set for Sn-Pt/HTCMG70 catalyst, C_3H_8:H_2O = 80%:20%, P = 5 bar.

Reaction	Kinetic parameters		
Propane Dehydrogenation	Parameter	UOM	Value
R1 $C_3H_8 \rightleftharpoons C_3H_6 + H_2$	k_{01}	mmol / (g*min*bar)	0.626692 ± 0.014
	E_{a1}	kJ/mol	72.230 ± 1.62
	K_0	-	4427 ± 98.99
	ΔH	kJ/mol	-79.998 ± 1.7888
Propane Cracking			
R2 $C_3H_8 \rightarrow C_2H_4 + CH_4$	k_{02}	mmol/(g*min*bar)	$0.00212 \pm 4.74 * 10^{-5}$
	E_{a2}	kJ/mol	301.865 ± 6.75

Ethylene hydrogenation			
R3 $C_2H_4 + H_2 \rightarrow C_2H_6$	k_{03}	mmol/(g*min*bar^2)	140.4 ± 3.14
	E_{a3}	kJ / mol	200.00 ± 4.47
Coke formation			
R4 $C_3H_6 \rightarrow 3CH_{0.5} + 2.25\ H_2$	k_{01C}	$mg_{cat}/(mg_{coke}$ * min)	5.68 *10^{-14} ± 1.27 * 10^{-15}
	E_{a1C}	kJ/mol	40.352 ± 0.902
	k_{02C}	$mg_{coke}/(mg_{cat}$ * min)	7.50 *10^{-3} ± 1.67 * 10^{-4}
	E_{a2C}	kJ/mol	53.568± 1.197
	C_{max}	mg_{coke}/mg_{cat}	0.000494 ± 1.1 * 10^{-5}

Table S4: Calculated OF function values for the three different deactivation models.

Deactivation model	OF
D1	**1.5976**
D2	**0.9944**
D3	**1.5944**
